# The infection staging and profile of genotypic distribution and drug resistance mutation among the human immunodeficiency virus-1 infected blood donors from five Chinese blood centers, 2012–2014

**DOI:** 10.1371/journal.pone.0179328

**Published:** 2017-06-16

**Authors:** Peibin Zeng, Yu Liu, Miao He, Jingxing Wang, Sheila Keating, Wei Mao, Mei Huang, Hongli Ma, Weilan He, Xinhong Bi, Dan Liao, Michael Busch, Paul Ness, Jing Liu, Hua Shan, on behalf of the NHLBI Recipient Epidemiology and Donor Evaluation Study-III program

**Affiliations:** 1 West China School of Public Health, Sichuan University, Chengdu, Sichuan, China; 2 Institute of Blood Transfusion, Chinese Academic of Medical Science, Chengdu, Sichuan, China; 3 Blood System Research Institute, San Francisco, California, United States of America; 4 Chongqing Blood Center, Chongqing, China; 5 Mianyang Blood Center, Mianyang, Sichuan, China; 6 Louyang Blood Center, Luoyang, Henan, China; 7 Guangxi Blood Center, Liuzhou, Guangxi, China; 8 Xinjiang Blood Center, Urumqi, Xinjiang, China; 9 Research Triangle Institute, Research Triangle Park, North Carolina, United States of America; 10 Department of Transfusion Medicine, Johns Hopkins Hospital, Baltimore, Maryland, United States of America; 11 Department of Pathology, Stanford University, Palo Alto, California, United States of America; Harvard Medical School, UNITED STATES

## Abstract

The increasing complexity and diversity of the human immunodeficiency virus-1 (HIV-1) infections challenge the disease control and anti-retrovirus treatment in China. The infection stages and molecular characteristics of HIV-1 from infected Chinese blood donors were examined to shed light on the HIV genotype distribution and the status of drug resistance mutations (DRMs) in the changing HIV epidemic in China. Western blot (WB) confirmed HIV-1 positive plasma samples were collected from blood donors at five Chinese blood centers from April 16, 2012, through June 30, 2014. The HIV infection stages were determined using the Lag-avidity assay. HIV *Pol* regions including whole protease and partial reverse transcriptase (RT) were amplified and sequenced to establish the profile of genotype distribution and drug resistance mutations (DRMs). Viral loads were determined using the ROCHE COBAS system. Of the 259 HIV-1 positive samples tested by the Lag-avidity assay, 23.6% (61/259) were identified as recent infections. A total of 205 amplified sequences displayed the following genotype distributions: circulating recombinant form (CRF) 07_BC (61.5%), CRF08_BC (8.3%), CRF01_AE (20%), B (6.3%), and 01B (3.9%). There was no significant difference in genotype distribution between recent and long-term infections. 31 DRMs were identified from 27 samples including four protease inhibitors (PIs) accessory DRMs, two PIs major DRMs (M46I), two nucleoside RT inhibitors DRMs (K219R and K70Q), and 23 nonnucleoside RT inhibitors DRMs. 27 samples had DRMs, yielding a drug resistance prevalence of 13.2% (27/205). Our findings provide important information for developing strategies for comprehensive HIV control and improving anti-retroviral treatment in China.

## Introduction

Accordign to updated Chinese government report, by the end of 2014, there were approximately 501,000 people living with human immunodeficiency virus Type 1 (HIV-1) (295,358 receiving anti-retroviral treatment) and 159,000 reported HIV related deaths in China [1]. The same report also estimated that there is large number of undiagnosed HIV infections due to the lack of awareness for HIV infection risk and accessibility to HIV testing especially in underdeveloped regions [1]. Studies indicated that the epidemic of HIV-1 had been spreading from high risk groups into the general population including blood donors in China[2–4].

The genotypic characteristics and profile of drug resistance mutations (DRMs) of HIV may provide substantial information to monitor trends of the viral evolution, and to optimize treatment strategy when initiating anti-retroviral treatment (ART). Understanding the genomic diversity of variant HIV strains is also critical to improve blood donor screening assays in order to prevent unknown infected donations from getting into the blood supply, especially in a rapidly evolving and changing HIV epidemic. In China, the major subtypes of HIV-1 in the general population include: circulating recombinant form (CRF) 07_BC, CRF08_BC, CRF01_AE and subtype B, while HIV-2 is seldom reported[5, 6]. Data on the current subtypes of HIV-1 infections among Chinese infected blood donors are limited. Previous research reported distinctive regional differences in subtype distribution among HIV infected blood donors in 1980’s. For example, former paid blood donors from Henan and other central China provinces were mostly of subtype B [7, 8]. In Kunming in Southwest China, from 2005–2006, a study found that of 49 specimens from infected blood donors, the distribution of HIV subtypes were: CRF08_BC(51.0%), CRF07_BC(24.5%), CRF01_AE (20.4%) and B(4.1%)[9]. In our previous Retrovirus Epidemiology Donor Study-II (REDS-II) funded by US National Heart Lung and Blood Institute (NHLBI), we reported the following HIV-1 genotype distribution among HIV infected blood donors from five Chinese blood centers (Kunming, Liuzhou, Urumqi, Luoyang and Mianyang) during 2007 to 2010: G (0.9%), B (2.7%), CRF01_AE (32.7%), CRF07_BC (22.1%), and CRF08_BC (41.6%)[10]. The drug resistance rate among HIV-1 infections was 4.4% in the same study.

Classification of the HIV recent and long-term (or chronic) infection by laboratory detection of biological markers is an important tool for accurate estimation of HIV incidence. Despite the potential, unavoidable misclassifications[11], several serological assays designed to identify the HIV infection stages have made contributions to the global HIV prevention and control, such as: the BED capture enzyme immunoassay (BED CEIA)[12], Vironostika-LS[13], Avidity-AxSym Gu[14], and the Limiting Antigen Avidity EIA (LAg-Avidity EIA)[15]. Early identification of new HIV infections is not only essential to initiate timely treatment and prevent further spread among individuals but also critical to understand the current trend of transmission, identify high-risk populations and risk factors, and monitor prevention efforts to effectively reduce transmissions in the general population[16]. Yet up to date, there is little data on assay-based HIV incidence testing to identify HIV recent infections among Chinese blood donors.

The HIV molecular surveillance among blood donors was launched as a part of the Recipient Epidemiology and Donor Evaluation Study (REDS-III) funded by the US NHBLI since 2012. In this study, we performed multiple laboratory-based testing to determine the HIV infection stages, genotype diversity and characteristics of HIV-1 DRMs among infected blood donors from five RED-III Chinese blood centers (Chongqing, Liuzhou, Urumqi, Luoyang and Mianyang). The implications of possible correlation between infection stages (e.g. recent/long-term infection), HIV genotypes and DRMs were investigated. Such information can be combined with data from the general Chinese population and high risk populations to help improve HIV screening strategies and safeguard the blood supply in China.

## Materials and methods

This study was approved by institutional review board (IRB) of Johns Hopkins Medicine, NA_00080591/ CR00012868 S1 Text and ethical review committee of Chinese Academy of Medical Sciences/Pekin Union Medical College, X101222002 S1 Fig.

### Study samples

From April 2012 to June 2014, a total of 265 HIV western blot (WB) confirmed positive donor samples were collected from five REDS-III blood centers: Chongqing (n = 153), Urumqi (n = 53), Liuzhou (n = 31), Luoyang (n = 17) and Mianyang (n = 11). The annual donations from five blood centers were approximately: Chongqing, 100,000; Liuzhou, 65,000; Urumqi, 40,000; Luoyang, 75,000; and Mianyang, 45,000 respectively. During the study period, approximate 90% HIV screening reactive samples underwent confirmatory testing and all available samples with confirmatory testing results from these five blood centers were included in this study. Routine parallel enzyme-linked immunosorbent assay (ELISA) screening was performed on all donations at each blood center using two of the following six assays at each center: Kehua Bio-engineering Co. Ltd. Shanghai; KINGHWAWK PHARMACEUTICAL. Inc. Beijing; Wantai Biological Pharmacy Enterprise CO., LTD., Beijing; Biomerieux., Inc., Shanghai; ZHUHAI LIVZON DIAGNOSTIC., INC., Zhuhai; Bio-Rad, Inc., Hercules, CA). ELISA screening reactive plasma samples were then collected, frozen, and stored at −20°C at each blood center before being shipped in batches to the Chinese Institute of Blood Transfusion (IBT) via cold chain transportation for WB confirmatory testing (MP Diagnostics HIV BLOT 2·2, MP Biomedicals AsiaPacific Pte Ltd, Singapore). Written consents from donors were obtained to have their blood samples tested for research purposes at the time of donation.

### HIV infection stages identification

The single-well LAg-Avidity EIA (LAg-Avidity EIA, Sedia Biosciences CO., Portland. OR) was implemented to determine the HIV infection stages following the manufacturer’s specification. This assay classifies HIV infection stages into recent infection and long-term infection based on avidity of HIV antibodies[17]. It has good performance among different HIV subtypes and populations[15] and was approved by US CDC. The mean duration of recency infection classified by the assay is approximately 140 days as described in the inserted manual (HIV recent infections would be identified as being infected within about 140 days by testing, all others were long-term infections).

### Extraction, amplification, sequencing and viral load testing of HIV-1 RNA

Two methods were applied for HIV-1 RNA extraction, amplification and sequencing at IBT:

1. HIV-1 RNA was extracted from 350μL of plasma using a viral RNA isolation kit (MagMAX, Ambion, Inc., Austin, TX) and eluted to a 75μL suspension according to the manufacturer’s manual. Approximately 1022bp of the HIV-1 *pol* region including the entire protease gene (297 nucleotides encoding 99 amino acids) and partial reverse transcriptase gene (the first 241 amino acids) were amplified by reverse transcription nested polymerase chain reaction (RT-nest-PCR) using previously reported method[10, 18]. Both DNA strands of PCR products were purified and sequenced by a DNA sequencing company (BGI, Inc., Beijing, China).
2. According to the method using Abbott HIV-1 genotyping assay (ViroSeq™ HIV-1 Genotyping System v2.0., Abbott Molecular Inc., Des Plaines, U.S.), the plasma samples were extracted for HIV-1 RNA and underwent RT-PCR with RNA extraction and RT-PCR modules inserted in the assay package. The length of 1302 nucleotides HIV-1 *pol* segment including the whole protease gene and partial polymerase gene was amplified. The PCR products were purified and sequenced by AB 3730 DNA Analyzer (Applied Biosystems, Foster City, CA) with modules inserted in the Viroseq assay.

The HIV-1 viral load testing was performed using individual HIV-1 nucleic acid testing (NAT) assay (AmpliPrep-COBAS TaqMan HIV-1 Test Version 2·0, COBAS S201, Roche Diagnostics Ltd, Mannheim, Germany.). The lower limit of detection was 20 copies/ml, given by the manual of the assay.

### Genotype, phylogenetic and drug resistance mutation analysis

Sequences were edited and aligned by BioEdit, v7.0.4.1 (http://www.mbio.ncsu.edu/BioEdit/bioedit.html). The phylogenetic tree was built with tool of MEGA 6.06 (http://www.megasoftware.net) by the neighbor-joining method under Kimura’s two parameter correction (1000 replicates). The HIV-1 reference sequences were collected from the Los Alamos database (http://www.hiv.lanl.gov) as the following (Accession Number): CRF07_BC, AF503396, JF906665, HQ215583, JQ901027, JQ901094, EF122515; CRF01_AE, AY008714, U51189, JF906597, JQ028206; CRF08_BC, AF286229, AY008717; Subtype B, K03455, U71182; Subtype 01B, KF857447, AY358072, KC183783 and SIVcpz as an outgroup X52154.

The HIV subtypes were then determined by subtypes reference sets from the Los Alamos HIV-1 sequence database (http://www.hiv.lanl.gov). The DRMs were analyzed by submitting the sequences to the Stanford HIVdb Program Genotypic Resistance Interpretation Algorithm (http://hivdb.stanford.edu, HIVdb version 8.3, last updated: 2017-03-02). HIVdb is an expert system that accepts user-submitted HIV-1 protease, reverse transcriptase (RT) and integrase sequences or mutations and returns inferred levels of resistance to 22 FDA-approved anti-retroviral drugs including 8 protease inhibitors (PIs), 7 nucleoside RT inhibitors (NRTIs), 4 non-nucleoside RT inhibitors (NNRTIs), and 3 integrase strand transfer inhibitors (INSTIs). The HIVdb system assigns a drug penalty score and a comment to each HIV DRM; five levels of inferred drug resisitance: susceptible, potential low-level resistance (PLLR), low-level resistance (LLR), intermediate resistance (IR), and high-level resistance (HLR) are reported based on the total score derived by adding the scores of each DRM associated with resistance to the specific drug.

### Statistical analysis

Demographic information was extracted from the donor/donation database from each blood center, merged, cleaned, and maintained by the REDS-III program. Statistical analysis was performed using the statistical software SAS (SAS,Windows 9.4, SAS Institute, Cary, NC; 2016). Chi-square tests (or Fisher’s exact test if a cell in the cross table has fewer than 5 observations) were conducted on all testing outcomes by blood center, donor demographic categories, and long term vs. recent HIV infection. P<0.05 was considered statistically significant.

### Gene accession numbers

The HIV-1 sequences in the study can be retrieved from GenBank with accession numbers from KU954560 to KU954764 S2 Text.

## Results

**Fig 1:**
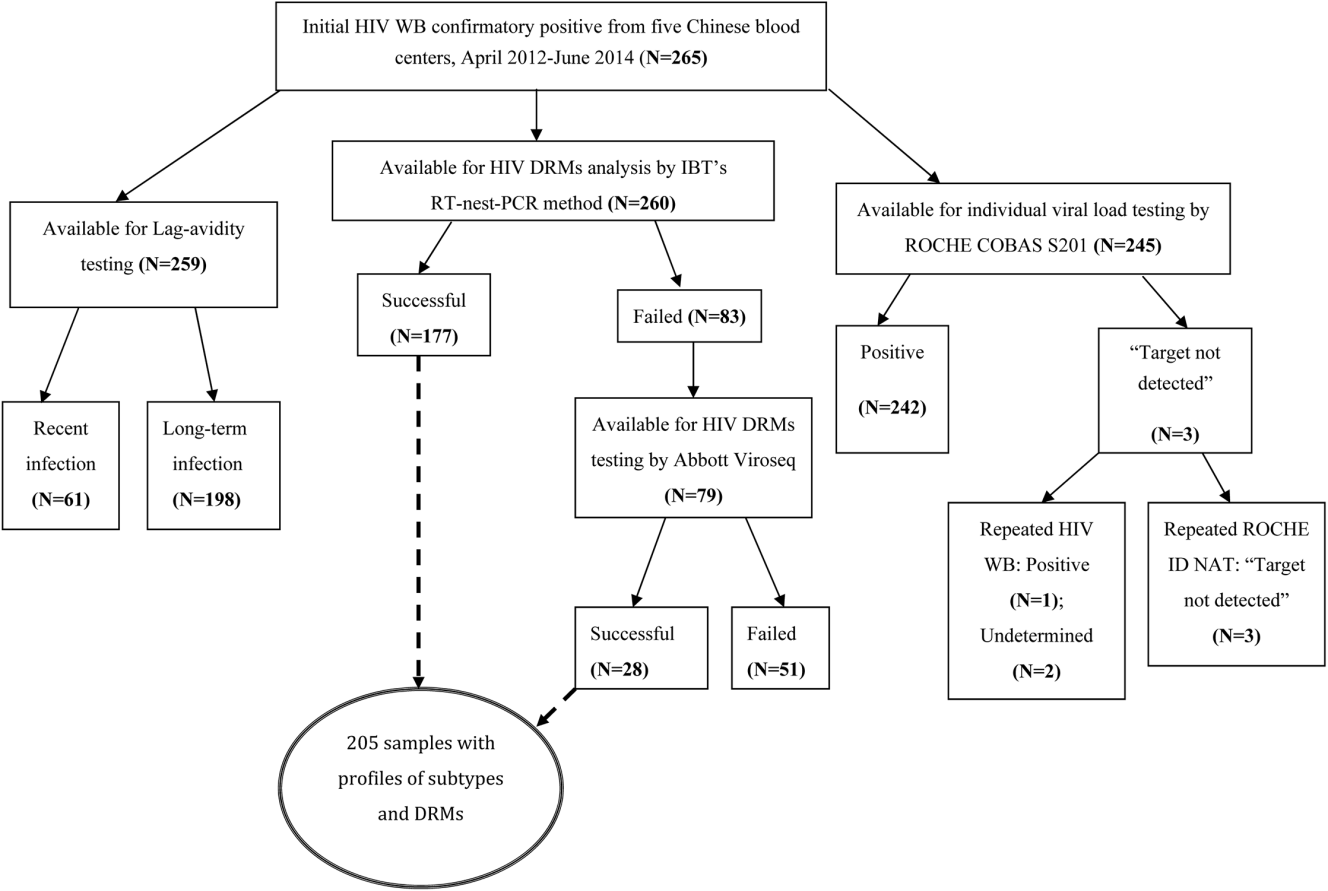
Summary of HIV testing and DRMs analysis among blood donors from five Chinese blood centers, April 2012 to June 2014.

Among 265 initial HIV confirmatory positive samples from blood donors enrolled in this study, 1) 259 were available for Lag-avidity testing to identify HIV infection stages; 2) 260 were available for amplification on *pol* region of HIV-1 for further genotyping and DRMs analysis; 3) 245 were available for HIV individual viral load testing. Summary of the HIV testing and analysis is displayed in Fig 1.

### HIV infection stages

Of 259 initial HIV confirmatory positive samples tested by Lag-avidity assay, 61(23.6%) were identified as recent infection and 198 (76.4%) as long-term infection (Table 1). The recency rates varied between 16.1% and 26.5% at the five blood centers without significant difference (P>0.05). Donor demographic characteristics were analyzed by recent and long-term infections but no significant difference was found by age, gender, ethnicity and occupation.

**Table 1:** Infection stages of 259 HIV infected donors from five Chinese blood centers. (N, %)^a^.

Blood centers	Chongqing	Urumqi	Guangxi	Luoyang	Mianyang	In total
Recent infection	40 (26.5%)	11 (20.8%)	5 (16.1%)	3 (17.6%)	2 (28.6%)	61 (23.6%)
Long-term infection	111 (73.5%)	42 (79.2%)	26 (83.9%)	14 (82.4%)	5 (71.4%)	198 (76.4%)
In total	151	53	31	17	7	259

^a ^Data are reported as number and percent of total within each blood center.

Of the 259 samples with Lag-avidity results, 55 recent infection and 179 long-term infection samples underwent viral load testing. The viral loads of recent infections (n = 55, 4300±17953 copies/ml) were lower than those of long-term infections (10300±53062 copies/ml) at the marginal level of statistical significance (P = 0.07). The proportion of repeat donors among recent infections was higher than that of long-term infections with no statistical significance (19.7%, 12/61 versus 10.6%, 21/198; P = 0.1). The 12 repeat donors with recent infection were 10 whole blood donors with an average inter-donation interval of 299.4±165.1 days (from their last negative donations) and two apheresis donors with 36 and 86 days inter-donation intervals.

### Demographic characteristics and HIV subtype distributions

Among 260 HIV confirmed positive samples available for amplification on *pol* region of HIV-1, 205 HIV partial *pol* sequences were amplified, sequenced and analyzed for HIV subtype. Among them, 177 were amplified using IBT’s RT-nest-PCR procedure and 28 samples that failed to be amplified by IBT’s procedure were successfully amplified using Abbott Viroseq assay (Fig 1). Demographic information of the 205 donors from five blood centers, including: Chongqing (n = 129), Urumqi (n = 29), Guangxi (n = 26), Luoyang (n = 13) and Mianyang (n = 8), is displayed in Table 2. Most of these donors were younger than 50 years old (97.6%). More males than females were enrolled and 12.7% were non-Han minority donors. More than half were first time donors (66.8%) and the two major occupations were “working at home” and “blue-collar worker”, accounting for 31.2% and 26.8% of all occupations respectively. The HIV recent infection rate among these 205 donors was 20% (41/205). A total of 51 samples failed to be amplified by either method. Amplification failure for these samples might be caused by their significantly lower viral loads (viral load = 1270±11812 copies/ml) when compared to those successfully amplified (viral load = 13300±51155 copies/ml), p = 0.013.

**Table 2:** Demographic characteristics and subtypes of 205 infected donors whose HIV genotypes and DRMs were successfully analyzed.

Demographic Characteristics		Chongqing (N = 129)	Urumqi (N = 29)	Guangxi (N = 26)	Luoyang (N = 13)	Mianyang (N = 8)	Total (N = 205)
**Age (years/old)**	18–30	82 (63.6%)	14 (48.3%)	11 (42.3%)	4 (30.8%)	4 (50%)	105 (56.1%)
	31–50	44 (34.1%)	15 (51.7%)	14 (52.9%)	9 (69.3%)	3 (37.5%)	85 (41.5%)
	>50	3 (2.3%)	0	1 (3.8%)	0	1 (12.5%)	5 (2.4%)
**Gender**	Male	116 (89.9%)	25 (86.2%)	19 (73.1%)	12 (92.3%)	5 (62.5%)	177 (86.3%)
	Female	13 (10.1%)	4 (13.8%)	7 (26.9%)	1 (7.7%)	3 (37.5%)	28 (13.7%)
**Ethnicity**	Han	125 (96.9%)	20 (69%)	14 (53.8%)	13 (100%)	7 (87.5%)	179 (87.3%)
	Others	4 (3.1%)	9 (31%)	12 (46.2%)	0	1 (12.5%)	26 (12.7%)
**Occupation**	Student	18 (14%)	5 (17.2%)	0	2 (15.4%)	1 (12.5%)	26 (12.7%)
	Blue-collar worker	35 (27.1%)	13 (44.8%)	4 (15.4%)	2 (15.4%)	1 (12.5%)	55 (26.8%)
	Farmer	12 (9.3%)	2 (6.9%)	0	4 (30.8%)	1 (12.5%)	19 (9.3%)
	Working at Home	38 (29.5%)	5 (17.2%)	15 (57.7%)	3 (23.1%)	3 (37.5%)	64 (31.2%)
	Others	26 (20.2%)	4 (13.8%)	7 (26.9%)	2 (15.4%)	2 (25%)	41 (20%)
**Donation**	First-time Donor	87 (67.4%)	23 (79.3%)	17 (65.4%)	6 (46.2%)	4 (50%)	137 (66.8%)
	Repeat Donor	42 (32.6%)	6 (20.7%)	9 (34.6%)	7 (53.8%)	4 (50%)	68 (33.2%)
**Education**	Bachelor's/Master's	17 (13.2%)	6 (20.7%)	1 (3.8%)	3 (23.1%)	0	27 (13.2%)
	Associate/Technicians	35 (27.1%)	9 (31%)	7 (26.9%)	1 (7.7%)	2 (25%)	54 (26.3%)
	Technician						
	High School or below	72 (55.8%)	13 (44.8%)	16 (61.5%)	9 (69.2%)	6 (75%)	116 (56.6%)
	Other/missing	5 (3.9%)	1 (3.4%)	2 (7.7%)	0	0	8 (3.9%)
	Missing						
**HIV Subtype**	CRF07_BC	94 (72.9%)	20 (69%)	2 (7.7%)	4 (30.7%)	6 (75%)	126 (61.5%)
	CRF01_AE	13 (10.1%)	4 (13.8%)	19 (73.1%)	3 (23.1%)	2 (25%)	41 (20%)
	CRF08_BC	13 (10.1%)	0	4 (15.4%)	0	0	17 (8.3%)
	B	6 (4.6%)	3 (10.3%)	1 (3.8%)	3 (23.1%)	0	13 (6.3%)
	01B	3 (2.3%)	2 (6.9%)	0	3 (23.1%)	0	8 (3.9%)

**Fig 2:**
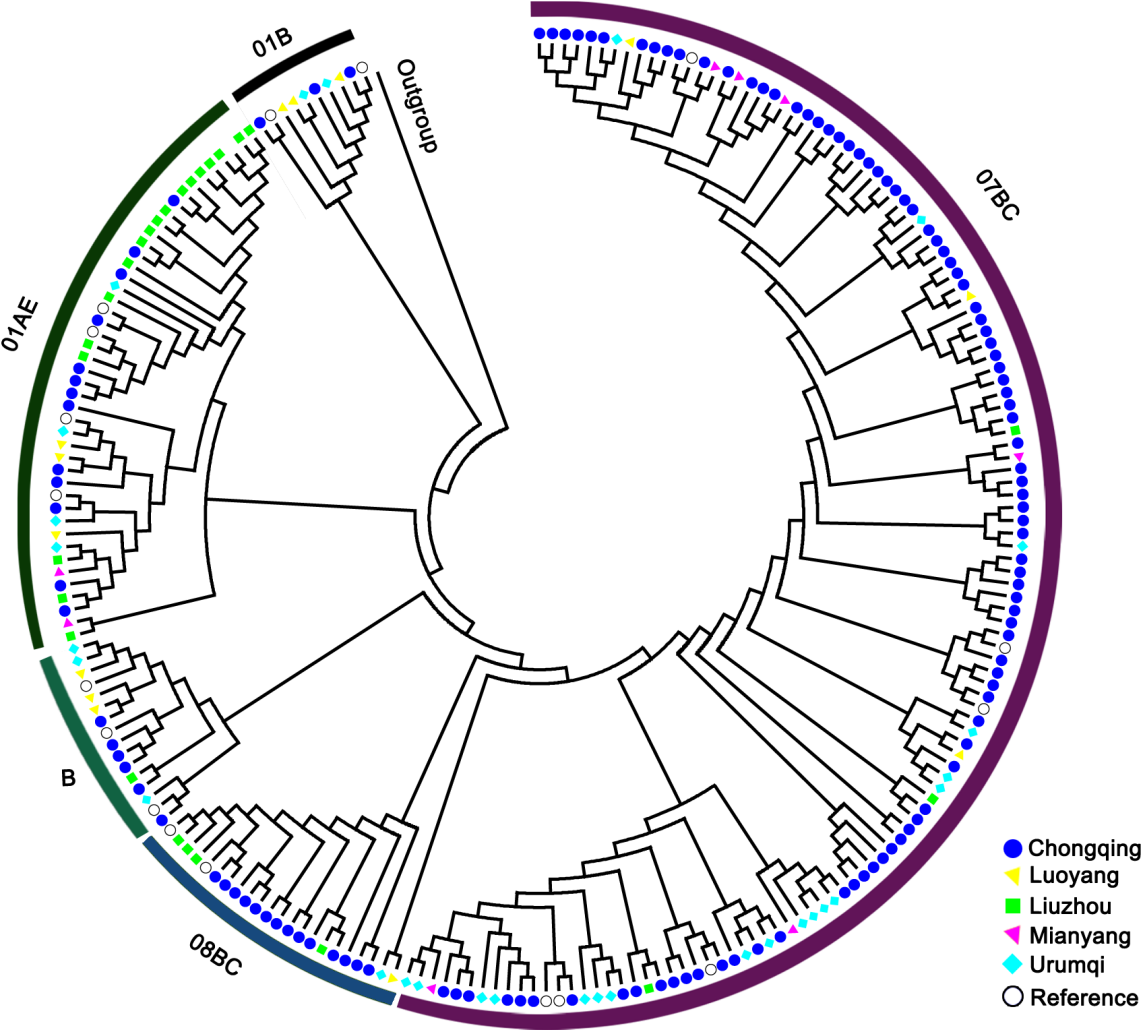
Phylogenic tree of the HIV-1 sequences.

As a whole group, the HIV-1 subtype by phylogenetic analysis was displayed as (Fig 2): CRF07_BC = 126 (61.5%), CRF08_BC = 17 (8.3%), CRF01_AE = 41 (20%), B = 13 (6.3%), and 01B = 8 (3.9%). CRF07_BC and CRF01_AE were two major subtypes. The HIV-1 subtype distribution in Chongqing was as follows: CRF07_BC = 94 (72.9%), CRF08_BC = 13 (10.1%), CRF01_AE = 13 (10.1%), B = 6 (4.6%), and 01B = 3 (2.3%). The dominant subtypes at Urumqi and Guangxi were CRF07_BC (69%, 20/29) and CRF01_AE (73.1%, 19/26) respectively. No significant difference of HIV-1 subtype’s distribution was observed between recent and long-term infections.

### Profile of HIV-1 genotypic drug resistance mutations

Of 205 samples analyzed, 27 were identified to bear HIV-1 drug resistance mutations (DRMs) S3 Text. The demographic characteristics of the 27 DRMs and 178 non-DRMs donors were analyzed, but no significant difference was found by age, gender, ethnicity, and other categories. 31 specific DRMs were identified from 27 samples with DRMs distributing as: 1) 74.2% (23/31) DRMs were on nonnucleoside reverse transcriptase inhibitors (NNRTIs) as: V179E/D (n = 16), K238N (n = 2), E138A/G (n = 4) and G190E (n = 1). 2) Six protease inhibitors (PIs) DRMs were observed including, four PIs accessory DRMs: L23I (n = 1), K43T (n = 1) and Q58E (n = 2); and two PIs major DRMs: M46I. 3) Two nucleotide reverse transcriptase inhibitors (NRTIs) DRMs were: K219R and K70Q.

Twenty-seven samples were interpreted as having HIV-1 drug resistance (DR) with potential low-level resistance (PLLR) or above and the rate of HIV-1 DR rate was 13.2% (27/205). The characteristics of donors with DR were shown in Table 3. The rates of HIV-1 DR varied by blood centers with the following distribution: Chongqing (13.2%, 17/129), Liuzhou (0%, 0/26), Urumqi (20.7%, 6/29), Luoyang (23.1%, 3/13), and Mianyang (12.5%, 1/8). Most of these DR donors were male with long-term HIV infections (23/27) at a mean age of 30.6 ± 9.1 (Median ±SD) years old. Different levels of DR to multiple drugs were observed among these 27 donors. For example, 16 of 27 donors showed PLLR (or above) to NNRTI due to V179D/E mutations; three donors with mutations of E138A were found to have PLLR to etravirine (ETR) and low-level resistance (LLR) to rilpivirine (RPV); specimen CQ12003214 was identified to have PLLR to five PIs and intermediate resistance (IR) to nelfinavir (NFV) caused by PI major DRM of M46I; two samples had PLLR to nelfinavir (NFV) and LLR to tipranavir (TPV) due to PI accessory DRM of Q58E; While one male donor (Specimen ID: LY12004233) from Luoyang with HIV recent infection bears high-level resistance (HLR) to efavirenz (EFV), nevirapine (NVP) and RPV and intermediate-level resistance (ILR) to ETR due to mutation of E138G and G190E.

**Table 3:** Characteristics of donors with drug resistance of potential low-level resistance or above (N = 27).

Specimen ID	Gender	Age in years^a^ (Median = 30.6±9.1)	Infection stages	Donations^b^	Genotyping	PI accessory DRMs (n = 4)	PI major DRMs (n = 2)	NRTI DRMs (n = 2)	NNRTI DRMs (n = 23)	Drug resistance^c^
CQ12000196	Male	26	LONG-TERM	First time	07_BC	L23I				LLR to NFV
CQ12002493	Male	40	LONG-TERM	First time	08_BC				V179E	PLLR to EFV, ETR, RPV and NVP
CQ12003214	Male	52	LONG-TERM	First time	07_BC		M46I			PLLR to ATV, FPV, IDV, LPV and SQV; IR to NFV
CQ12003677	Male	40	LONG-TERM	First time	07_BC			K219R	K238N	PLLR to AZT, D4T, EFV and NVP
CQ12003834	Male	30	LONG-TERM	First time	07_BC				V179D	PLLR to EFV, ETR, RPV and NVP
cq12004073	Male	18	RECENT	First time	01B				V179E	PLLR to EFV, ETR, RPV and NVP
cq12004297	Male	33	LONG-TERM	First time	08_BC				V179D	PLLR to EFV, ETR, RPV and NVP
cq12004326	Male	24	LONG-TERM	Repeat	07_BC				V179D	PLLR to EFV, ETR, RPV and NVP
cq12004551	Male	44	RECENT	First time	08_BC				E138A	PLLR to ETR; LLR to RPV
CQ13000959	Male	41	RECENT	Repeat	08_BC				E138A	PLLR to ETR; LLR to RPV
CQ13001022	Male	32	LONG-TERM	Repeat	08_BC				E138A	PLLR to ETR; LLR to RPV
CQ13001278	Male	18	LONG-TERM	First time	07_BC			K70Q		PLLR to FTC and 3TC; LLR to ABC, D4T, DDI, and TDF
CQ13001603^d^	Male	20	LONG-TERM	First time	07_BC				V179D	PLLR to ETR; LLR to RPV; IR to EFV and NVP
CQ13002447	Female	38	LONG-TERM	First time	07_BC				V179D	PLLR to EFV, ETR, RPV and NVP
CQ13002852	Male	34	LONG-TERM	Repeat	07_BC				K238N	PLLR to EFV and NVP
CQ13003037	Male	26	LONG-TERM	First time	01B				V179E	PLLR to EFV, ETR, RPV and NVP
CQ13003228	Male	18	LONG-TERM	First time	07_BC	Q58E				PLLR to NFV; LLR to TPV
LY12003986	Male	22	LONG-TERM	Repeat	01B				V179E	PLLR to EFV, ETR, RPV and NVP
LY12004233	Male	32	RECENT	First time	07_BC				E138G and G190E	HLR to EFV, NVP and RPV; ILR to ETR
LY14003600	Male	39	LONG-TERM	Repeat	01B				V179E	PLLR to EFV, ETR, RPV and NVP
MY14000058	Female	30	LONG-TERM	First time	07_BC	Q58E				PLLR to NFV; LLR to TPV
WS12000312	Male	23	LONG-TERM	First time	01B				V179E	PLLR to EFV, ETR, RPV and NVP
WS12000334	Male	25	LONG-TERM	First time	B	K43T	M46I		V179E	PLLR to ATV, FPV, IDV, LPV and SQV; LLR to TPV; IR to NFV; PLLR to EFV, ETR, RPV and NVP
WS12000352	Male	26	LONG-TERM	First time	07_BC				V179D	PLLR to EFV, ETR, RPV and NVP
ws12001012	Male	20	LONG-TERM	First time	01_AE				V179D	PLLR to EFV, ETR, RPV and NVP
WS12001503	Male	38	LONG-TERM	First time	01B				V179E	PLLR to EFV, ETR, RPV and NVP
WS13003163	Male	38	LONG-TERM	First time	07_BC				V179D	PLLR to EFV, ETR, RPV and NVP

a. Median age denoted as mean followed by SD

b. The time from last negative donation in six repeat donors: 373.5 ±90.7 days (mean ±SD).

c. As determined by the Stanford HIVdb Program Genotypic Resistance Interpretation Algorithm (http://hivdb.stanford.edu). Potential Low level Resistance (PLLR), Low Level Resistance (LLR), intermediate level resistance (ILR) or high-level resistance (HLR); NFV, Nelfinavir; EFV, efavirenz; ETR, etravirine; RPV, rilpivirine; NVP, nevirapine; ATV, atazanavir; FPV, Fosamprenavir; IDV, indinavir; LPV, lopinavir; AZT, Zidovudine; D4T, Stavudine; 3TC, Lamivudine; ABC, Abacavir; D4T, Sanilvudin; DDI, didanosine; FTC, Emtricitabine; TDF, Tenofovir disoproxil fumarate; TPV, tipranavir; SQV, saquinavir.

d. The combination of V179D and K103R (not defined as NNRTI DRM) act synergistically to reduce NVP and EFV susceptibility.

### Three serologically positive but viral loads not detected (VLND) samples

Of 245 samples that underwent individual HIV NAT, 242 were individual NAT positive with viral loads ranging between: viral loads<20 copies/ml and 5.3×10^5^ copies/ml, and 3 were repeated viral loads not detected (VLND) (Fig 1). After repeat HIV WB testing on these VLND samples, 2 of 3 were WB undetermined. The characteristics of these VLND samples were displayed in Table 4. One of these three samples (Specimen ID: WS13003483) was from a repeat donor who made a negative donation 86 days before. Two WB repeat test undetermined samples were only P24 positive in repeat WB testing. One was EIA screening grey zone (0.8≤S/Co<1.0). The other was parallel EIA reactive sample with P31 and P55 negative at initial and repeat WB testing.

**Table 4:** Three Serologically positive but viral load not detected samples^a^.

Specimen ID	Lag-avidity	Parallel EIA screening, R1 (S/Co)	Parallel EIA screening, R2 (S/Co)	Initial WB Banding patterns^c^	Repeat WB Banding patterns
CQ12004687	RECENT	Neg(0.06)	Grey zone^b^ (0.8)	gp160 (+), gp120 (+) and P24 (+); The rest negative	P24 (+); The rest negative
CQ-12-004896	RECENT	Neg (0.05)	Pos (1.1)	gp160 (+), gp120 (+) and P24 (+); The rest negative	P24 (+); The rest negative
WS13003483^d^	RECENT	Pos(>1.0)	Pos (>1.0)	P55 (-) and P31(-); The rest positive	P55 (-) and P31(-); The rest positive

^a^, Viral load not detected: Repeated individual NAT using ROCHE COBAS S201 platform, resulting as “Target not detected”. All these three samples failed to be amplified by both RT-nest-PCR methods.

^b^, EIA grey zone: 0.8≤S/Co<1.0

^c^, Confirmatory testing at IBT used WB assay: MP Diagnostics HIV BLOT 2.2 (MP Biomedicals Asia Pacific Pte Ltd, Singapore). The ten bands in the assay are: gp160, gp120, p66, p55, p51, gp41, p31, p24, p17 and HIV-2. All these samples were negative on HIV-2. The criteria calling WB positive was: 1) At least two positive on gp160, gp 120 and gp41; 2) At least one positive on the three in 1) while P24 positive.

^d^, Repeat donor with the time from last negative donation being 86 days.

## Discussion

The rate of HIV recent infections varied worldwide due to differences in HIV prevalence, infection risks, and the use of different laboratory detection assays[16]. In this study, the rate of HIV recent infections among infected blood donors based on Lag Avidity testing was 23.6% (61/259), which was similar to that among HIV seropositive donors in Brazil during 2007 to 2011[19] (17.5%, 43/246) and that among HIV nucleic acid testing (NAT) positive donors in the U.S during 2006 to 2009 [20] (23.6%, 210/891,), where incidence testing assay of Vironostika-LS was used. The recency rate was also similar to the proportion of newly diagnosed infections in Zhejiang province, China (21.8%, 46/211, BED CEIA)[21]. The recency rate among blood donors was lower than those in the high-risk populations in China such as men who have sex with men (MSM) (32.8%, 261/795, LAg-avidity EIA) from 14 cities, [22] in Hebei province (40.7%, 50/123)[23], Tianjin (34.1%, 45/132)[24] and Henan province (42.3%, 11/26)[25] using BED CEIA assay; but higher than in intravenous drug users (IDUs) in Yunnan province (7.1% to 9.7%, BED CEIA) [26, 27]. The HIV recency rate among infected donors is reflective of the transmission patterns among the high-risk populations. In China, there was a trend of increasing HIV infection risk among MSM[21], such as in Chongqing where an HIV prevalence rate as high as 18.7% was found among MSM [28]. Also, 27.2% of newly identified HIV infections in China were from MSM from January to October 2015 and the infection rate is still rising in this high-risk group [29]. Based on these different observations, we hypothesize that test-seeking from high-risk individuals is likely a contributing factor to the recent infections among the population of HIV infected donors in our study. Future studies on the risk behaviors of infected blood donors are needed to confirm this hypothesis.

In our study, samples with recent infections bear relatively lower viral loads than those with long-term infections, perhaps because low-level viremia may appear in early primary HIV-1 infections among blood donors[30]. The complication of viral loads and early primary HIV infection should be further categorized into different stages accompanied by panels of longitudinal samples[31]. Unfortunately such longitudinal samples were not available in this study for further exploration. According to the government guideline for blood center operation in China[32], the time intervals for whole blood (400 ml) and apheresis platelet donation should be no less than six months and two weeks respectively. As a matter of fact, more than 90% of all donations in this study were whole blood donations, and the 6-month mandatory inter-donation interval for whole blood donors was much longer than the duration of recent infection (e.g. <140 days) that can be captured by LAg-avidity EIA, which may blur the difference of recency rate between repeat and first-time donors.

Blood donors in this study were from regions with the highest HIV prevalence rates in China, including four blood centers located in vast western China and one (Luoyang) in central region. The most frequently observed subtypes among blood donors from western China were subtype CRF07_BC (61.5%) and CRF01_AE (20%), which was different than the HIV subtypes found in our previous REDS-II study with two major subtypes CRF08_BC (41.6%) and CRF01_AE (32.7%)[10]. Most of the samples in previous REDS-II study (82/113) were collected in Kunming that contributed more than half of the major subtype CRF08_BC (57.3%) infections. Whereas in the present REDS-III study, 129 of 205 samples were from Chongqing with CRF07_BC (72.9%) as the dominate subtype. Chongqing was one of key transportation hubs on the drug traffic route from Yunnan province, where Kunming is the capital, to Xinjiang province in western China[33], so it is not surprising that CRF_BC was as dominant in Chongqing as in Kunming, Chengdu, and regions of Xinjiang province, but not in other regions of China. For example, most of the subtypes in central China (e.g. Henan province) are subtype B[8] and the major subtype at southeastern and eastern coastal area is subtype 01_AE.[21, 34, 35] The variations in subtype distribution might be due to different transmission patterns and status of co-evolution between virus and population, which couldn’t be confirmed in this study. The main subtypes found at Urumqi and Liuzhou blood centers: CRF07_BC and CRF01_AE, were the same as in our previous REDS-II findings[10], which were also consistent with the subtype distributions among high-risks groups at these two regions[36, 37]. Previous studies reported that most HIV-1 infected individuals from Henan province (Louyang is located in Henan province) were subtype B infected via unsafe blood collection (mostly former paid plasma collection)[38]. In our study, 13 samples from Luoyang were a mixture of subtypes CRF07_BC, CRF01_AE, B and 01B. If we assume that the subtype distribution in infected donors matches that in the local high risk populations, then there might be an increasing complexity of HIV-1 transmission routes in Luoyang in recent years.

The DRMs prevalence rate reported here (13.2%, 27/205) was lower than the published results from other studies about Chinese blood donors or anti-retroviral-naive populations that varied from 17% to 37.8%[10, 18, 39, 40]. Perhaps a result that some polymorphic accessory mutations such as: A71V/T and L10I/V on PIs; V106I and V90I on NNRTIs, commonly observed in previous studies[10, 41], were no longer classified as PI minor DRMs and NNRTI resistance mutations in the updated Stanford HIVdb Program Genotypic Resistance Interpretation Algorithm (HIVdb version 8.3).

The prevalence of HIV-1 DR (13.2%) was similar to those reported in studies performed in the United States (9.9%[42] and 10.9%[43]), European countries (14.2%)[44] and recent finding among Chinese former paid donors in Henan province (17.7%, 19/109)[41]; but higher than those from other Chinese HIV-1 treatment naïve populations in Yunnan province (2% to 4.3%)[10, 39, 45], Hangzhou (4%)[21], Jiangsu (2.1% to 4.1%)[35], Shaanxi (4.4%)[46] and Chengdu (1.3%)[18]; and also higher than those from other high risk populations such as: MSM (2%-5.3%)[23, 47–49], IDU (3.8%-4.4%) in China. In Urumqi, the HIV-1 DR was 24.1% (7/29), which was different than the previous REDS-II study that found no sample harboring HIV-1 DR (0/14) in the same region during 2007 to 2010 [10]. In the current study, most of samples had DR due to DRMs on NNRTIs (23/31). Drug-specific NNRTIs mutations such as: V179E, K238N and G190E were associated with the selection in patients receiving Nevirapine (NVP) and Efavirenz (EFV) according to Stanford HIVdb program. These two drugs were mostly adopted as first-line NNRTIs for anti-retrovirus treatment (ART) in China[50].While V179D and E138A mutations were described as polymorphic accessory NNRTI-selected mutation, however, it is unclear whether the V179D and E138A mutations have resulted from drug-specific or natural selective pressure by Chinese population. According to the Chinese National Free AIDS Antiretroviral Therapy Manual, PIs are not included as the first-line ART drugs in China. Q58E and M46I mutations, interpreted by the Stanford HIVdb program as non-polymorphic PI-selected mutations, were probably not obtained from domestic transmissions. If we assume that most of the HIV infected donors were not aware of the infection status at donation and didn’t receive HIV ART, then some DRMs observed in the present study could be transmitted from the treated HIV infected population, especially for the donor with high drug resistance to NNRTIs due to mutations of G190E and E138A.This hypothesis is yet to be confirmed with more data in future studies.

In addition, three serologically positive but VLND samples were identified in this study. However, the western blots in these three cases each lacked p31 reactivity. It has been reported that this pattern (p31 negative and HIV-1 RNA negative) can indicate false positive western blot results[51]. Therefore, we believe these three samples were likely to be western blot false positive. However, we did not collect longitudinal samples to further verify this interpretation.

There are several limitations in this study including: 1) The unbalanced number of HIV infected donors from five blood centers may make it difficult to compare the status of HIV-1 genotypic distribution and DRMs between each site; 2) The length of study period and sample size were limited. More samples should be included to obtain more comprehensive profile of HIV infections among blood donors in China in our future efforts; 3) Information of the HIV infection risk factors from infected donors is limited and urgently awaited to help on illuminating the relations between HIV transmission routes, HIV-1 genotype diversity, as well as profile of HIV DRMs and drug resistance. 4) Full genome sequences of HIV strains from infected donors should be obtained to give more comprehensive HIV genomic information for genotyping and evaluation on the virus evolution. However, our results may provide an important contemporary picture of HIV infections among donors in parts of China with comparatively high prevalence of HIV infections, especially regions with increasing infection risk of MSM.

In conclusion, our results on the recency rate of HIV infection among Chinese blood donors reminds us the importance of timely surveillance and update on information of HIV risk factors among donors. The increasing prevalence of HIV-1 DR among Chinese donors compared to our previous REDS-II study indicates the urgent need to improve HIV antiviral therapeutic strategy and implement comprehensive HIV control programs in China.

## Supporting information

S1 Fig IRB from CAMS. (JPG)

S1 Text IRB from JHM. (PDF)

S2 Text 205 sequences from Chinese HIV-1 infected donors. (TXT)

S3 Text HIVdb report on donors with potential low DR or above (n = 27). (PDF)
