# Supplementary figures and images for: The infection staging and profile of genotypic distribution and drug resistance mutation among the human immunodeficiency virus-1 infected blood donors from five Chinese blood centers, 2012–2014
Source: PLoS One. 2017 Jun 16;12(6):e0179328. doi: 10.1371/journal.pone.0179328 (PMC5473534; doi:10.1371/journal.pone.0179328)

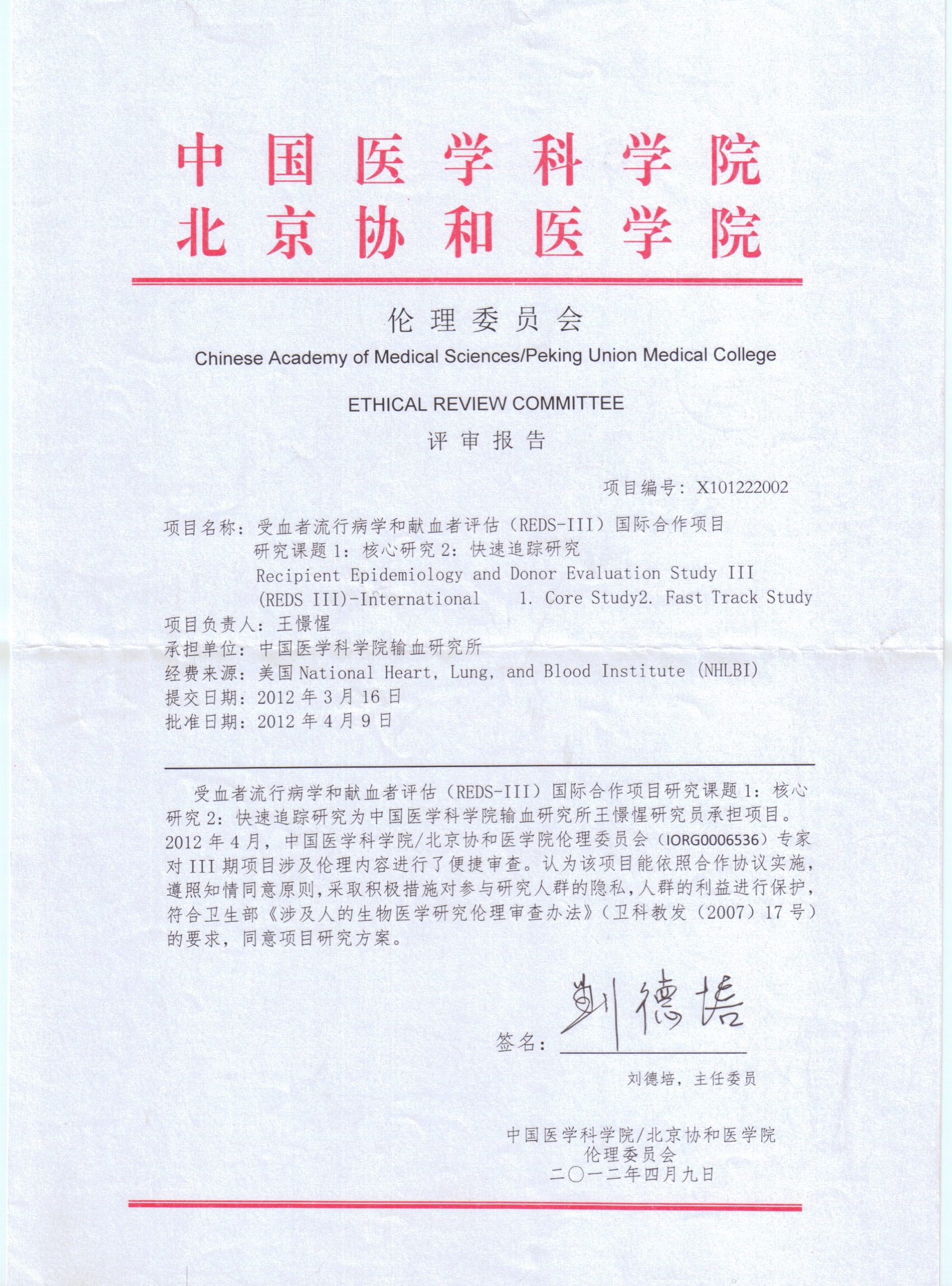

Supplement: S1 Fig — (JPG) [file pone.0179328.s001.jpg]
