# Supplementary material for: The infection staging and profile of genotypic distribution and drug resistance mutation among the human immunodeficiency virus-1 infected blood donors from five Chinese blood centers, 2012–2014
Source: PLoS One. 2017 Jun 16;12(6):e0179328. doi: 10.1371/journal.pone.0179328 (PMC5473534; doi:10.1371/journal.pone.0179328)
Supplement: S1 Text — (PDF) [file pone.0179328.s002.pdf]

JOHNS HOPKINS
MEDICINE

**Office of Human Subjects Research**
***Institutional Review Boards***

1620 McElderry Street, Reed Hall, Suite B-130
Baltimore, Maryland 21205-1911
410-955-3008
410-955-4367 Fax
e-mail: jhmirb@jhmi.edu

**Date:** November 3, 2016

# CONTINUING REVIEW APPROVAL

| | |
|---|---|
| **Review Type:** | Expedited |
| **Principal Investigator:** | Hua Shan |
| **Number:** | NA_00080591 / CR00012868 |
| **Title:** | HIV molecular surveillance studies |
| **Committee Chair:** | Susan Bassett |
| **IRB Committee:** | IRB-X |

**Date of approval:** November 3, 2016

**Date of Expiration:** November 2, 2017

The JHM IRB approved the above-referenced Continuing Review.

This is a retrospective study and data collection is ongoing.

If this study is a clinical trial and data collection is complete for the perspecified primary outcome, Section 801 of the Food and Drug Administration Amendments Act requires reporting of summary results information at http://www.clinicaltrials.gov. Reporting must be done within 12 months of completing data collection for the prespecified primary outcome, regardless of sponsor or funding source. Failure to comply with this law may result in civil penalties. For more information on results reporting go to http://www.clinicaltrials.gov. If the study is registered with Clinicaltrials.gov and is closed to recruitment and enrollment, the record must be updated within 30 days to reflect the study's enrollment status. See http://clinicaltrials.gov/ct2/manage-recs/how-edit for more information. Questions can be directed to register@clinicaltrials.gov.

**Date of Approval and Expiration Date:** The approval and expiration date for this research are listed above. If the approval lapses, the research must stop and you must submit a request to the IRB to determine whether it is in the best interests of individual participants to continue with protocol-related procedures.

**Changes in Research:** All proposed changes to the research must be submitted using a Change in Research application. The changes must be approved by the JHM IRB prior to implementation, with the following exception: changes made to eliminate apparent immediate hazards to participants may be made immediately, and promptly reported to the JHM IRB.

**Continuing Review:** Continuing Review Applications should be submitted at least 6 weeks prior to the study expiration date. Failure to allow sufficient time for review may result in a lapse of approval. If the Continuing Review Application is not submitted prior to the expiration date, your study will be terminated and a New Application must be submitted to reinitiate the research.

**Unanticipated Problems:** All unanticipated problems must be submitted using a Protocol Event Report.

If this research has a commercial sponsor, the research may not start until the sponsor and JHU have signed a contract.

The Johns Hopkins Institutions operates under multiple Federal-Wide Assurances: The Johns Hopkins University School of Medicine - FWA00005752, The Johns Hopkins University School of Nursing - FWA00006088, The Johns Hopkins Hospital and Johns Hopkins Health Systems - FWA00006087, Johns Hopkins Bayview Medical Center - FWA00006089, Howard County General Hospital - FWA00005743, Hugo W. Moser Research Institute at Kennedy Krieger, Inc. - FWA00005719, Johns Hopkins Community Physicians - FWA00002251, Suburban Hospital and Health System - FWA00005924
