# Supplementary material for: The infection staging and profile of genotypic distribution and drug resistance mutation among the human immunodeficiency virus-1 infected blood donors from five Chinese blood centers, 2012–2014
Source: PLoS One. 2017 Jun 16;12(6):e0179328. doi: 10.1371/journal.pone.0179328 (PMC5473534; doi:10.1371/journal.pone.0179328)
Supplement: S3 Text — (PDF) [file pone.0179328.s004.pdf]

Stanford University
HIV DRUG RESISTANCE DATABASE

HIVdb Program Report

Genotypic Resistance Interpretation Algorithm

HIVdb version 8.3 (last updated 2017-03-02)

Sequence CQ12000196

Sequence Summary

Sequence includes PR:	codons 1 - 99
Sequence includes RT:	codons 1 - 241
Subtype:	CRF07_BC (2.65%)
PR SDRMs:	L23I
RT SDRMs:	None

Sequence Quality Assessment

PR

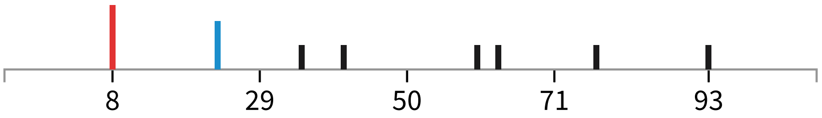

RT

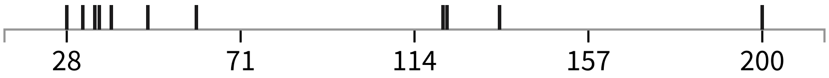

Drug Resistance Interpretation: PR

PI Major Resistance Mutations:	None
PI Accessory Resistance Mutations:	**L23I**
Other Mutations:	R8L, E35D, R41K, D60E, L63P, V77I, I93L

Protease Inhibitors

**atazanavir/r (ATV/r)**	Susceptible
**darunavir/r (DRV/r)**	Susceptible
**fosamprenavir/r (FPV/r)**	Susceptible
**indinavir/r (IDV/r)**	Susceptible
**lopinavir/r (LPV/r)**	Susceptible
**nelfinavir (NFV)**	Low-Level Resistance
**saquinavir/r (SQV/r)**	Susceptible
**tipranavir/r (TPV/r)**	Susceptible

PR Comments

PI Accessory

- **L23I** is an uncommon non-polymorphic mutation selected primarily by NFV. It causes low-level NFV resistance.

Mutation Scoring: PR

PI	ATV/r	DRV/r	FPV/r	IDV/r	LPV/r	NFV	SQV/r	TPV/r
L23I	0	0	0	0	0	15	0	0
Total	0	0	0	0	0	15	0	0

Drug Resistance Interpretation: RT

NRTI Resistance Mutations:	None
NNRTI Resistance Mutations:	None
Other Mutations:	E28K, K32E, V35T, E36A, T39D, S48T, V60I, D121H, K122E, I135T, T200I

Nucleoside Reverse Transcriptase Inhibitors

**abacavir (ABC)**	Susceptible
**zidovudine (AZT)**	Susceptible
**stavudine (D4T)**	Susceptible
**didanosine (DDI)**	Susceptible
**emtricitabine (FTC)**	Susceptible

**lamivudine (3TC)**	Susceptible
**tenofovir (TDF)**	Susceptible

Non-nucleoside Reverse Transcriptase Inhibitors

**efavirenz (EFV)**	Susceptible
**etravirine (ETR)**	Susceptible
**nevirapine (NVP)**	Susceptible
**rilpivirine (RPV)**	Susceptible

Mutation Scoring: RT

NRTI	ABC	AZT	D4T	DDI	FTC	3TC	TDF
Total	0	0	0	0	0	0	0

NNRTI	EFV	ETR	NVP	RPV
Total	0	0	0	0

Sequence CQ12002493

Sequence Summary

Sequence includes PR:	codons 1 - 99
Sequence includes RT:	codons 1 - 241
Subtype:	B + C (3.53%)
PR SDRMs:	None
RT SDRMs:	None

Sequence Quality Assessment

PR

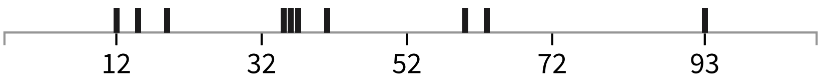

RT

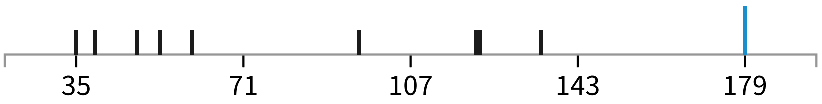

Drug Resistance Interpretation: PR

PI Major Resistance Mutations:	None
PI Accessory Resistance Mutations:	None
Other Mutations:	T12P, I15V, L19I, E35D, M36I, N37H, R41K, D60E, L63P, I93L

Protease Inhibitors	
**atazanavir/r (ATV/r)**	Susceptible
**darunavir/r (DRV/r)**	Susceptible
**fosamprenavir/r (FPV/r)**	Susceptible
**indinavir/r (IDV/r)**	Susceptible
**lopinavir/r (LPV/r)**	Susceptible
**nelfinavir (NFV)**	Susceptible
**saquinavir/r (SQV/r)**	Susceptible

**tipranavir/r (TPV/r)**	Susceptible

Mutation Scoring: PR

PI	ATV/r	DRV/r	FPV/r	IDV/r	LPV/r	NFV	SQV/r	TPV/r
Total	0	0	0	0	0	0	0	0

Drug Resistance Interpretation: RT

NRTI Resistance Mutations:	None
NNRTI Resistance Mutations:	**V179E**
Other Mutations:	V35T, T39D, S48T, E53D, V60I, H96P, D121Y, K122E, I135R

Nucleoside Reverse Transcriptase Inhibitors

**abacavir (ABC)**	Susceptible
**zidovudine (AZT)**	Susceptible
**stavudine (D4T)**	Susceptible
**didanosine (DDI)**	Susceptible
**emtricitabine (FTC)**	Susceptible
**lamivudine (3TC)**	Susceptible
**tenofovir (TDF)**	Susceptible

Non-nucleoside Reverse Transcriptase Inhibitors

**efavirenz (EFV)**	Potential Low-Level Resistance
**etravirine (ETR)**	Potential Low-Level Resistance
**nevirapine (NVP)**	Potential Low-Level Resistance
**rilpivirine (RPV)**	Potential Low-Level Resistance

RT Comments

NNRTI

- V179D is a polymorphic accessory NNRTI-selected mutation. It contributes low-levels reductions in susceptibility to each of the NNRTIs. The combination of V179D and K103R act synergistically to reduce NVP and EFV susceptibility. V179D has a low weight in the Tibotec ETR genotypic susceptibility score. **V179E** is a non-polymorphic mutation occasionally selected by NVP and EFV. **V179E** appears similar to V179D in its

effects on NNRTIs. **V179D/E** do not appear to reduce the virological response to a first-line EFV-containing regimen.

Mutation Scoring: RT

NRTI	ABC	AZT	D4T	DDI	FTC	3TC	TDF
Total	0	0	0	0	0	0	0

NNRTI	EFV	ETR	NVP	RPV
V179E	10	10	10	10
Total	10	10	10	10

Sequence CQ12003214

Sequence Summary

Sequence includes PR:	codons 1 - 99
Sequence includes RT:	codons 1 - 241
Subtype:	B + C (3.04%)
PR SDRMs:	M46I
RT SDRMs:	None

Sequence Quality Assessment

PR

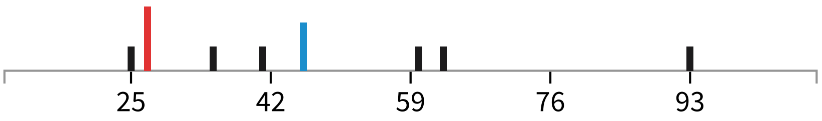

RT

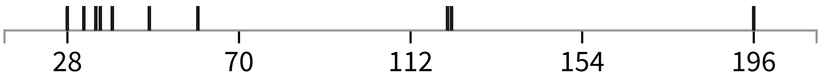

Drug Resistance Interpretation: PR

PI Major Resistance Mutations:	**M46I**
PI Accessory Resistance Mutations:	None
Other Mutations:	D25Y, G27R, E35D, R41K, D60E, L63P, I93L

Protease Inhibitors

**atazanavir/r (ATV/r)**	Potential Low-Level Resistance
**darunavir/r (DRV/r)**	Susceptible
**fosamprenavir/r (FPV/r)**	Potential Low-Level Resistance
**indinavir/r (IDV/r)**	Potential Low-Level Resistance
**lopinavir/r (LPV/r)**	Potential Low-Level Resistance
**nelfinavir (NFV)**	Intermediate Resistance
**saquinavir/r (SQV/r)**	Potential Low-Level Resistance

tipranavir/r (TPV/r) Susceptible

PR Comments

PI Major

- **M46I/L** are relatively non-polymorphic PI-selected mutations. In combination with other PI-resistance mutations, they are associated with reduced susceptibility to each of the PIs except DRV.

Mutation Scoring: PR

PI	ATV/r	DRV/r	FPV/r	IDV/r	LPV/r	NFV	SQV/r	TPV/r
M46I	10	0	10	10	10	30	10	5
Total	10	0	10	10	10	30	10	5

Drug Resistance Interpretation: RT

NRTI Resistance Mutations: None
NNRTI Resistance Mutations: None
Other Mutations: E28K, K32E, V35T, E36A, T39D, S48T, V60I, D121H, K122E, G196R

Nucleoside Reverse Transcriptase Inhibitors

abacavir (ABC) Susceptible
zidovudine (AZT) Susceptible
stavudine (D4T) Susceptible
didanosine (DDI) Susceptible
emtricitabine (FTC) Susceptible
lamivudine (3TC) Susceptible
tenofovir (TDF) Susceptible

Non-nucleoside Reverse Transcriptase Inhibitors

efavirenz (EFV) Susceptible
etravirine (ETR) Susceptible
nevirapine (NVP) Susceptible
rilpivirine (RPV) Susceptible

Mutation Scoring: RT

NRTI	ABC	AZT	D4T	DDI	FTC	3TC	TDF
Total	0	0	0	0	0	0	0

NNRTI	EFV	ETR	NVP	RPV
Total	0	0	0	0

Sequence CQ12003677

Sequence Summary

Sequence includes PR:	codons 1 - 99
Sequence includes RT:	codons 1 - 241
Subtype:	CRF07_BC (2.45%)
PR SDRMs:	None
RT SDRMs:	K219R

Sequence Quality Assessment

PR

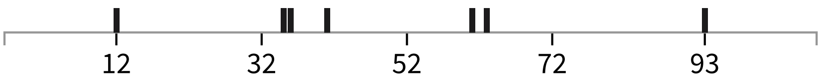

RT

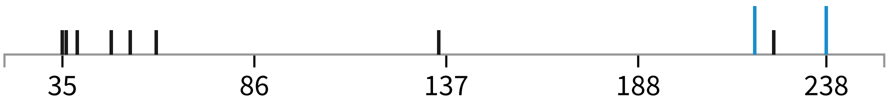

Drug Resistance Interpretation: PR

PI Major Resistance Mutations:	None
PI Accessory Resistance Mutations:	None
Other Mutations:	T12A, E35D, M36I, R41K, Q61H, L63P, I93L

Protease Inhibitors	
**atazanavir/r (ATV/r)**	Susceptible
**darunavir/r (DRV/r)**	Susceptible
**fosamprenavir/r (FPV/r)**	Susceptible
**indinavir/r (IDV/r)**	Susceptible
**lopinavir/r (LPV/r)**	Susceptible
**nelfinavir (NFV)**	Susceptible
**saquinavir/r (SQV/r)**	Susceptible
**tipranavir/r (TPV/r)**	Susceptible

Mutation Scoring: PR

PI	ATV/r	DRV/r	FPV/r	IDV/r	LPV/r	NFV	SQV/r	TPV/r
Total	0	0	0	0	0	0	0	0

Drug Resistance Interpretation: RT

NRTI Resistance Mutations:	**K219R**
NNRTI Resistance Mutations:	**K238N**
Other Mutations:	V35T, E36A, T39D, S48T, E53D, V60I, I135V, E224D

Nucleoside Reverse Transcriptase Inhibitors

**abacavir (ABC)**	Susceptible
**zidovudine (AZT)**	Potential Low-Level Resistance
**stavudine (D4T)**	Potential Low-Level Resistance
**didanosine (DDI)**	Susceptible
**emtricitabine (FTC)**	Susceptible
**lamivudine (3TC)**	Susceptible
**tenofovir (TDF)**	Susceptible

Non-nucleoside Reverse Transcriptase Inhibitors

**efavirenz (EFV)**	Potential Low-Level Resistance
**etravirine (ETR)**	Susceptible
**nevirapine (NVP)**	Potential Low-Level Resistance
**rilpivirine (RPV)**	Susceptible

RT Comments

NRTI

- **K219N/R** are accessory TAMS that usually occur in combination with multiple other TAMs.

NNRTI

- K238T is a non-polymorphic mutation selected in patients receiving NVP and EFV. It usually occurs in combination with K103N. Alone it confers low/intermediate reductions in NVP and EFV susceptibility. **K238N** is a non-polymorphic accessory mutation that is also selected by NVP and EFV. It appears to have minimal, if any, effect on NNRTI susceptibility.

Mutation Scoring: RT

NRTI	ABC	AZT	D4T	DDI	FTC	3TC	TDF
K219R	5	10	10	5	0	0	5
Total	5	10	10	5	0	0	5

NNRTI	EFV	ETR	NVP	RPV
K238N	10	0	10	0
Total	10	0	10	0

Sequence CQ12003834

Sequence Summary

Sequence includes PR:	codons 1 - 99
Sequence includes RT:	codons 1 - 241
Subtype:	CRF07_BC (2.75%)
PR SDRMs:	None
RT SDRMs:	None

Sequence Quality Assessment

PR

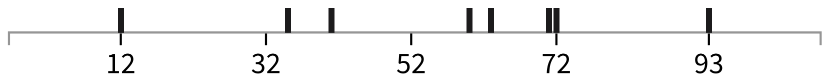

RT

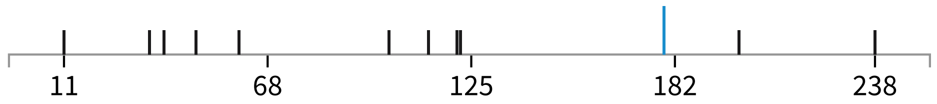

Drug Resistance Interpretation: PR

PI Major Resistance Mutations:	None
PI Accessory Resistance Mutations:	None
Other Mutations:	T12A, E35D, R41K, D60E, L63P, A71V, I72T, I93L

Protease Inhibitors	
**atazanavir/r (ATV/r)**	Susceptible
**darunavir/r (DRV/r)**	Susceptible
**fosamprenavir/r (FPV/r)**	Susceptible
**indinavir/r (IDV/r)**	Susceptible
**lopinavir/r (LPV/r)**	Susceptible
**nelfinavir (NFV)**	Susceptible
**saquinavir/r (SQV/r)**	Susceptible
**tipranavir/r (TPV/r)**	Susceptible

PR Comments

Other

- **A71V/T** are polymorphic, PI-selected accessory mutations that increase the replication of viruses with other PI-resistance mutations.

Mutation Scoring: PR

PI	ATV/r	DRV/r	FPV/r	IDV/r	LPV/r	NFV	SQV/r	TPV/r
Total	0	0	0	0	0	0	0	0

Drug Resistance Interpretation: RT

NRTI Resistance Mutations:	None
NNRTI Resistance Mutations:	**V179D**
Other Mutations:	K11N, V35T, T39N, S48T, V60I, K102R, D113N, D121H, K122E, T200I, K238R

Nucleoside Reverse Transcriptase Inhibitors

**abacavir (ABC)**	Susceptible
**zidovudine (AZT)**	Susceptible
**stavudine (D4T)**	Susceptible
**didanosine (DDI)**	Susceptible
**emtricitabine (FTC)**	Susceptible
**lamivudine (3TC)**	Susceptible
**tenofovir (TDF)**	Susceptible

Non-nucleoside Reverse Transcriptase Inhibitors

**efavirenz (EFV)**	Potential Low-Level Resistance
**etravirine (ETR)**	Potential Low-Level Resistance
**nevirapine (NVP)**	Potential Low-Level Resistance
**rilpivirine (RPV)**	Potential Low-Level Resistance

RT Comments

NNRTI

- **V179D** is a polymorphic accessory NNRTI-selected mutation. It contributes low-levels reductions in susceptibility to each of the NNRTIs. The combination of **V179D** and K103R act synergistically to reduce NVP and EFV susceptibility. **V179D** has a low weight in the Tibotec ETR genotypic susceptibility score. V179E is a non-polymorphic mutation occasionally selected by NVP and EFV. V179E appears similar to **V179D** in its effects on NNRTIs. **V179D/E** do not appear to reduce the virological response to a first-line EFV-containing regimen.

Other

- **K238R** is a common polymorphism that does not reduce NNRTI susceptibility.

Mutation Scoring: RT

NRTI	ABC	AZT	D4T	DDI	FTC	3TC	TDF
Total	0	0	0	0	0	0	0

NNRTI	EFV	ETR	NVP	RPV
V179D	10	10	10	10
Total	10	10	10	10

Sequence cq12004073

Sequence Summary

Sequence includes PR:	codons 1 - 99
Sequence includes RT:	codons 1 - 241
Subtype:	CRF55_01B (2.94%)
PR SDRMs:	None
RT SDRMs:	None

Sequence Quality Assessment

PR

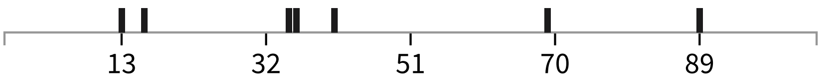

RT

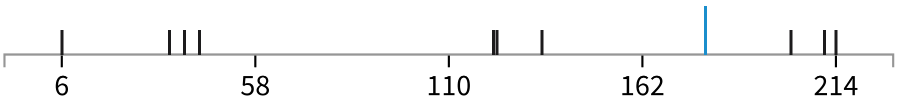

Drug Resistance Interpretation: PR

PI Major Resistance Mutations:	None
PI Accessory Resistance Mutations:	None
Other Mutations:	I13V, G16E, E35D, M36I, R41K, H69K, L89M

Protease Inhibitors	
**atazanavir/r (ATV/r)**	Susceptible
**darunavir/r (DRV/r)**	Susceptible
**fosamprenavir/r (FPV/r)**	Susceptible
**indinavir/r (IDV/r)**	Susceptible
**lopinavir/r (LPV/r)**	Susceptible
**nelfinavir (NFV)**	Susceptible
**saquinavir/r (SQV/r)**	Susceptible
**tipranavir/r (TPV/r)**	Susceptible

Mutation Scoring: PR

PI	ATV/r	DRV/r	FPV/r	IDV/r	LPV/r	NFV	SQV/r	TPV/r
Total	0	0	0	0	0	0	0	0

Drug Resistance Interpretation: RT

NRTI Resistance Mutations:	None
NNRTI Resistance Mutations:	**V179E**
Other Mutations:	E6D, V35T, T39K, K43E, K122E, D123S, I135R, I202V, R211K, F214L

Nucleoside Reverse Transcriptase Inhibitors

**abacavir (ABC)**	Susceptible
**zidovudine (AZT)**	Susceptible
**stavudine (D4T)**	Susceptible
**didanosine (DDI)**	Susceptible
**emtricitabine (FTC)**	Susceptible
**lamivudine (3TC)**	Susceptible
**tenofovir (TDF)**	Susceptible

Non-nucleoside Reverse Transcriptase Inhibitors

**efavirenz (EFV)**	Potential Low-Level Resistance
**etravirine (ETR)**	Potential Low-Level Resistance
**nevirapine (NVP)**	Potential Low-Level Resistance
**rilpivirine (RPV)**	Potential Low-Level Resistance

RT Comments

NNRTI

- V179D is a polymorphic accessory NNRTI-selected mutation. It contributes low-levels reductions in susceptibility to each of the NNRTIs. The combination of V179D and K103R act synergistically to reduce NVP and EFV susceptibility. V179D has a low weight in the Tibotec ETR genotypic susceptibility score. **V179E** is a non-polymorphic mutation occasionally selected by NVP and EFV. **V179E** appears similar to V179D in its effects on NNRTIs. **V179D/E** do not appear to reduce the virological response to a first-line EFV-containing regimen.

Mutation Scoring: RT

NRTI	ABC	AZT	D4T	DDI	FTC	3TC	TDF
Total	0	0	0	0	0	0	0

NNRTI	EFV	ETR	NVP	RPV
V179E	10	10	10	10
Total	10	10	10	10

Sequence cq12004297

Sequence Summary

Sequence includes PR:	codons 1 - 99
Sequence includes RT:	codons 1 - 241
Subtype:	CRF08_BC (2.55%)
PR SDRMs:	None
RT SDRMs:	None

Sequence Quality Assessment

PR

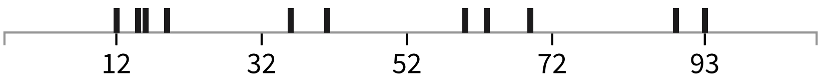

RT

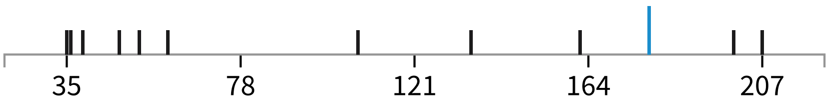

Drug Resistance Interpretation: PR

PI Major Resistance Mutations:	None
PI Accessory Resistance Mutations:	None
Other Mutations:	T12S, I15V, G16E, L19I, M36V, R41K, D60E, L63T, H69K, L89M, I93L

Protease Inhibitors	
**atazanavir/r (ATV/r)**	Susceptible
**darunavir/r (DRV/r)**	Susceptible
**fosamprenavir/r (FPV/r)**	Susceptible
**indinavir/r (IDV/r)**	Susceptible
**lopinavir/r (LPV/r)**	Susceptible
**nelfinavir (NFV)**	Susceptible
**saquinavir/r (SQV/r)**	Susceptible

**tipranavir/r (TPV/r)**	Susceptible

Mutation Scoring: PR

PI	ATV/r	DRV/r	FPV/r	IDV/r	LPV/r	NFV	SQV/r	TPV/r
Total	0	0	0	0	0	0	0	0

Drug Resistance Interpretation: RT

NRTI Resistance Mutations:	None
NNRTI Resistance Mutations:	**V179D**
Other Mutations:	V35T, E36A, T39E, S48T, E53D, V60I, T107S, I135V, S162C, T200A, Q207E

Nucleoside Reverse Transcriptase Inhibitors

**abacavir (ABC)**	Susceptible
**zidovudine (AZT)**	Susceptible
**stavudine (D4T)**	Susceptible
**didanosine (DDI)**	Susceptible
**emtricitabine (FTC)**	Susceptible
**lamivudine (3TC)**	Susceptible
**tenofovir (TDF)**	Susceptible

Non-nucleoside Reverse Transcriptase Inhibitors

**efavirenz (EFV)**	Potential Low-Level Resistance
**etravirine (ETR)**	Potential Low-Level Resistance
**nevirapine (NVP)**	Potential Low-Level Resistance
**rilpivirine (RPV)**	Potential Low-Level Resistance

RT Comments

NNRTI

- **V179D** is a polymorphic accessory NNRTI-selected mutation. It contributes low-levels reductions in susceptibility to each of the NNRTIs. The combination of **V179D** and K103R act synergistically to reduce NVP and EFV susceptibility. **V179D** has a low weight in the Tibotec ETR genotypic susceptibility score. V179E is a non-polymorphic mutation occasionally selected by NVP and EFV. V179E appears similar to **V179D** in its

effects on NNRTIs. **V179D/E** do not appear to reduce the virological response to a first-line EFV-containing regimen.

Mutation Scoring: RT

NRTI	ABC	AZT	D4T	DDI	FTC	3TC	TDF
Total	0	0	0	0	0	0	0

NNRTI	EFV	ETR	NVP	RPV
V179D	10	10	10	10
Total	10	10	10	10

Sequence cq12004326

Sequence Summary

Sequence includes PR:	codons 1 - 99
Sequence includes RT:	codons 1 - 241
Subtype:	CRF07_BC (2.65%)
PR SDRMs:	None
RT SDRMs:	None

Sequence Quality Assessment

PR

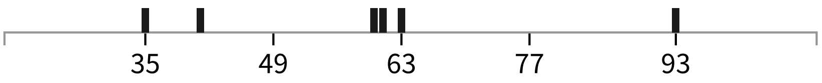

RT

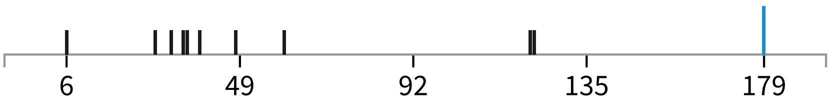

Drug Resistance Interpretation: PR

PI Major Resistance Mutations:	None
PI Accessory Resistance Mutations:	None
Other Mutations:	E35D, R41K, D60E, Q61E, L63P, I93L

Protease Inhibitors	
**atazanavir/r (ATV/r)**	Susceptible
**darunavir/r (DRV/r)**	Susceptible
**fosamprenavir/r (FPV/r)**	Susceptible
**indinavir/r (IDV/r)**	Susceptible
**lopinavir/r (LPV/r)**	Susceptible
**nelfinavir (NFV)**	Susceptible
**saquinavir/r (SQV/r)**	Susceptible
**tipranavir/r (TPV/r)**	Susceptible

Mutation Scoring: PR

PI	ATV/r	DRV/r	FPV/r	IDV/r	LPV/r	NFV	SQV/r	TPV/r
Total	0	0	0	0	0	0	0	0

Drug Resistance Interpretation: RT

NRTI Resistance Mutations:	None
NNRTI Resistance Mutations:	**V179D**
Other Mutations:	E6K, E28K, K32E, V35T, E36A, T39N, S48T, V60I, D121Y, K122E

Nucleoside Reverse Transcriptase Inhibitors

**abacavir (ABC)**	Susceptible
**zidovudine (AZT)**	Susceptible
**stavudine (D4T)**	Susceptible
**didanosine (DDI)**	Susceptible
**emtricitabine (FTC)**	Susceptible
**lamivudine (3TC)**	Susceptible
**tenofovir (TDF)**	Susceptible

Non-nucleoside Reverse Transcriptase Inhibitors

**efavirenz (EFV)**	Potential Low-Level Resistance
**etravirine (ETR)**	Potential Low-Level Resistance
**nevirapine (NVP)**	Potential Low-Level Resistance
**rilpivirine (RPV)**	Potential Low-Level Resistance

RT Comments

NNRTI

- **V179D** is a polymorphic accessory NNRTI-selected mutation. It contributes low-levels reductions in susceptibility to each of the NNRTIs. The combination of **V179D** and K103R act synergistically to reduce NVP and EFV susceptibility. **V179D** has a low weight in the Tibotec ETR genotypic susceptibility score. V179E is a non-polymorphic mutation occasionally selected by NVP and EFV. V179E appears similar to **V179D** in its effects on NNRTIs. **V179D/E** do not appear to reduce the virological response to a first-line EFV-containing regimen.

Mutation Scoring: RT

NRTI	ABC	AZT	D4T	DDI	FTC	3TC	TDF
Total	0	0	0	0	0	0	0

NNRTI	EFV	ETR	NVP	RPV
V179D	10	10	10	10
Total	10	10	10	10

Sequence cq12004551

Sequence Summary

Sequence includes PR:	codons 1 - 99
Sequence includes RT:	codons 1 - 241
Subtype:	CRF08_BC (2.94%)
PR SDRMs:	None
RT SDRMs:	None

Sequence Quality Assessment

PR

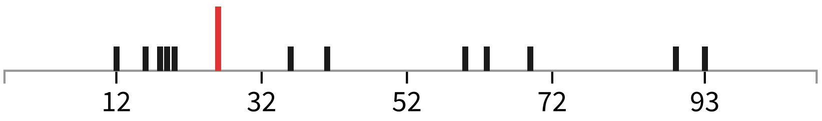

RT

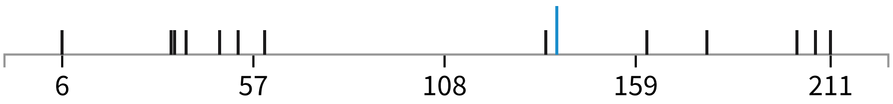

Drug Resistance Interpretation: PR

PI Major Resistance Mutations:	None
PI Accessory Resistance Mutations:	None
Other Mutations:	T12S, G16E, Q18P, L19I, K20R, T26R, M36I, R41K, D60E, L63V, H69K, L89M, I93L

Protease Inhibitors	
**atazanavir/r (ATV/r)**	Susceptible
**darunavir/r (DRV/r)**	Susceptible
**fosamprenavir/r (FPV/r)**	Susceptible
**indinavir/r (IDV/r)**	Susceptible
**lopinavir/r (LPV/r)**	Susceptible
**nelfinavir (NFV)**	Susceptible
**saquinavir/r (SQV/r)**	Susceptible

**tipranavir/r (TPV/r)**	Susceptible

PR Comments

Other

- **K20R** is a highly polymorphic PI-selected accessory mutation.

Mutation Scoring: PR

PI	ATV/r	DRV/r	FPV/r	IDV/r	LPV/r	NFV	SQV/r	TPV/r
Total	0	0	0	0	0	0	0	0

Drug Resistance Interpretation: RT

NRTI Resistance Mutations:	None
NNRTI Resistance Mutations:	**E138A**
Other Mutations:	E6D, V35T, E36A, T39D, S48T, E53D, V60I, I135V, S162C, I178L, I202V, Q207K, R211K

Nucleoside Reverse Transcriptase Inhibitors

**abacavir (ABC)**	Susceptible
**zidovudine (AZT)**	Susceptible
**stavudine (D4T)**	Susceptible
**didanosine (DDI)**	Susceptible
**emtricitabine (FTC)**	Susceptible
**lamivudine (3TC)**	Susceptible
**tenofovir (TDF)**	Susceptible

Non-nucleoside Reverse Transcriptase Inhibitors

**efavirenz (EFV)**	Susceptible
**etravirine (ETR)**	Potential Low-Level Resistance
**nevirapine (NVP)**	Susceptible
**rilpivirine (RPV)**	Low-Level Resistance

RT Comments

NNRTI

- **E138A** is a common polymorphic accessory mutation weakly selected in patients receiving ETR and RPV. It confers a borderline low-level reduction in RPV susceptibility. It has a low weight in the Tibotec ETR genotypic susceptibility score.

Mutation Scoring: RT

NRTI	ABC	AZT	D4T	DDI	FTC	3TC	TDF
Total	0	0	0	0	0	0	0

NNRTI	EFV	ETR	NVP	RPV
E138A	0	10	0	15
Total	0	10	0	15

Sequence CQ13000959

Sequence Summary

Sequence includes PR:	codons 1 - 99
Sequence includes RT:	codons 1 - 241
Subtype:	B + C (3.33%)
PR SDRMs:	None
RT SDRMs:	None

Sequence Quality Assessment

PR

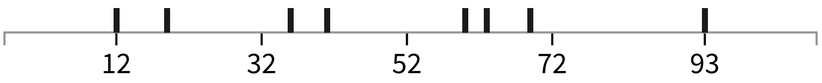

RT

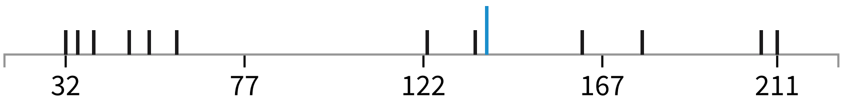

Drug Resistance Interpretation: PR

PI Major Resistance Mutations:	None
PI Accessory Resistance Mutations:	None
Other Mutations:	T12S, L19I, M36I, R41K, D60E, L63P, H69K, I93L

Protease Inhibitors	
**atazanavir/r (ATV/r)**	Susceptible
**darunavir/r (DRV/r)**	Susceptible
**fosamprenavir/r (FPV/r)**	Susceptible
**indinavir/r (IDV/r)**	Susceptible
**lopinavir/r (LPV/r)**	Susceptible
**nelfinavir (NFV)**	Susceptible
**saquinavir/r (SQV/r)**	Susceptible
**tipranavir/r (TPV/r)**	Susceptible

Mutation Scoring: PR

PI	ATV/r	DRV/r	FPV/r	IDV/r	LPV/r	NFV	SQV/r	TPV/r
Total	0	0	0	0	0	0	0	0

Drug Resistance Interpretation: RT

NRTI Resistance Mutations:	None
NNRTI Resistance Mutations:	**E138A**
Other Mutations:	K32R, V35T, T39D, S48T, E53D, V60I, D123E, I135V, S162C, D177E, Q207E, R211K

Nucleoside Reverse Transcriptase Inhibitors

**abacavir (ABC)**	Susceptible
**zidovudine (AZT)**	Susceptible
**stavudine (D4T)**	Susceptible
**didanosine (DDI)**	Susceptible
**emtricitabine (FTC)**	Susceptible
**lamivudine (3TC)**	Susceptible
**tenofovir (TDF)**	Susceptible

Non-nucleoside Reverse Transcriptase Inhibitors

**efavirenz (EFV)**	Susceptible
**etravirine (ETR)**	Potential Low-Level Resistance
**nevirapine (NVP)**	Susceptible
**rilpivirine (RPV)**	Low-Level Resistance

RT Comments

NNRTI

- **E138A** is a common polymorphic accessory mutation weakly selected in patients receiving ETR and RPV. It confers a borderline low-level reduction in RPV susceptibility. It has a low weight in the Tibotec ETR genotypic susceptibility score.

Mutation Scoring: RT

NRTI	ABC	AZT	D4T	DDI	FTC	3TC	TDF
Total	0	0	0	0	0	0	0

NNRTI	EFV	ETR	NVP	RPV
E138A	0	10	0	15
Total	0	10	0	15

Sequence CQ13001022

Sequence Summary

Sequence includes PR:	codons 1 - 99
Sequence includes RT:	codons 1 - 241
Subtype:	B + C (3.33%)
PR SDRMs:	None
RT SDRMs:	None

Sequence Quality Assessment

PR

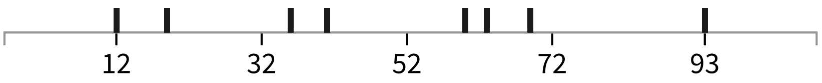

RT

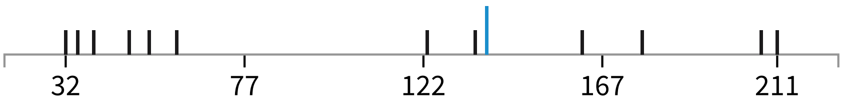

Drug Resistance Interpretation: PR

PI Major Resistance Mutations:	None
PI Accessory Resistance Mutations:	None
Other Mutations:	T12S, L19I, M36I, R41K, D60E, L63P, H69K, I93L

Protease Inhibitors	
**atazanavir/r (ATV/r)**	Susceptible
**darunavir/r (DRV/r)**	Susceptible
**fosamprenavir/r (FPV/r)**	Susceptible
**indinavir/r (IDV/r)**	Susceptible
**lopinavir/r (LPV/r)**	Susceptible
**nelfinavir (NFV)**	Susceptible
**saquinavir/r (SQV/r)**	Susceptible
**tipranavir/r (TPV/r)**	Susceptible

Mutation Scoring: PR

PI	ATV/r	DRV/r	FPV/r	IDV/r	LPV/r	NFV	SQV/r	TPV/r
Total	0	0	0	0	0	0	0	0

Drug Resistance Interpretation: RT

NRTI Resistance Mutations:	None
NNRTI Resistance Mutations:	**E138A**
Other Mutations:	K32R, V35T, T39D, S48T, E53D, V60I, D123E, I135V, S162C, D177E, Q207E, R211K

Nucleoside Reverse Transcriptase Inhibitors

**abacavir (ABC)**	Susceptible
**zidovudine (AZT)**	Susceptible
**stavudine (D4T)**	Susceptible
**didanosine (DDI)**	Susceptible
**emtricitabine (FTC)**	Susceptible
**lamivudine (3TC)**	Susceptible
**tenofovir (TDF)**	Susceptible

Non-nucleoside Reverse Transcriptase Inhibitors

**efavirenz (EFV)**	Susceptible
**etravirine (ETR)**	Potential Low-Level Resistance
**nevirapine (NVP)**	Susceptible
**rilpivirine (RPV)**	Low-Level Resistance

RT Comments

NNRTI

- **E138A** is a common polymorphic accessory mutation weakly selected in patients receiving ETR and RPV. It confers a borderline low-level reduction in RPV susceptibility. It has a low weight in the Tibotec ETR genotypic susceptibility score.

Mutation Scoring: RT

NRTI	ABC	AZT	D4T	DDI	FTC	3TC	TDF
Total	0	0	0	0	0	0	0

NNRTI	EFV	ETR	NVP	RPV
E138A	0	10	0	15
Total	0	10	0	15

Sequence CQ13001278

Sequence Summary

Sequence includes PR:	codons 1 - 99
Sequence includes RT:	codons 1 - 241
Subtype:	B + C (3.04%)
PR SDRMs:	None
RT SDRMs:	None

Sequence Quality Assessment

PR

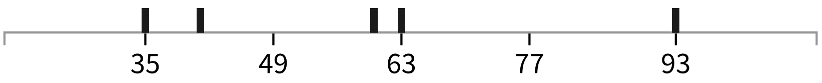

RT

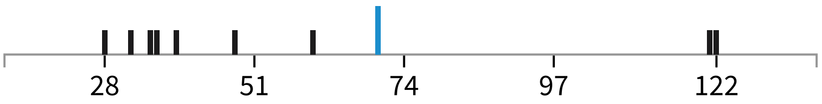

Drug Resistance Interpretation: PR

PI Major Resistance Mutations:	None
PI Accessory Resistance Mutations:	None
Other Mutations:	E35D, R41K, D60E, L63P, I93L

Protease Inhibitors	
**atazanavir/r (ATV/r)**	Susceptible
**darunavir/r (DRV/r)**	Susceptible
**fosamprenavir/r (FPV/r)**	Susceptible
**indinavir/r (IDV/r)**	Susceptible
**lopinavir/r (LPV/r)**	Susceptible
**nelfinavir (NFV)**	Susceptible
**saquinavir/r (SQV/r)**	Susceptible
**tipranavir/r (TPV/r)**	Susceptible

Mutation Scoring: PR

PI	ATV/r	DRV/r	FPV/r	IDV/r	LPV/r	NFV	SQV/r	TPV/r
Total	0	0	0	0	0	0	0	0

Drug Resistance Interpretation: RT

NRTI Resistance Mutations:	**K70Q**
NNRTI Resistance Mutations:	None
Other Mutations:	E28K, K32E, V35T, E36A, T39N, S48T, V60L, D121Y, K122E

Nucleoside Reverse Transcriptase Inhibitors

**abacavir (ABC)**	Low-Level Resistance
**zidovudine (AZT)**	Susceptible
**stavudine (D4T)**	Low-Level Resistance
**didanosine (DDI)**	Low-Level Resistance
**emtricitabine (FTC)**	Potential Low-Level Resistance
**lamivudine (3TC)**	Potential Low-Level Resistance
**tenofovir (TDF)**	Low-Level Resistance

Non-nucleoside Reverse Transcriptase Inhibitors

**efavirenz (EFV)**	Susceptible
**etravirine (ETR)**	Susceptible
**nevirapine (NVP)**	Susceptible
**rilpivirine (RPV)**	Susceptible

RT Comments

NRTI

- K70E/G cause low-level resistance to TDF, ABC, DDI and possibly 3TC and FTC. K70E/G increase susceptibility to AZT. **K70Q/N/S/T** are rare non-polymorphic NRTI-selected mutations that appear to have resistance profiles similar to K70E/G.

Mutation Scoring: RT

NRTI	ABC	AZT	D4T	DDI	FTC	3TC	TDF
K70Q	15	0	15	15	10	10	15
Total	15	0	15	15	10	10	15

NNRTI	EFV	ETR	NVP	RPV
Total	0	0	0	0

Sequence CQ13001603

Sequence Summary

Sequence includes PR:	codons 1 - 99
Sequence includes RT:	codons 1 - 241
Subtype:	B + C (3.33%)
PR SDRMs:	None
RT SDRMs:	None

Sequence Quality Assessment

PR

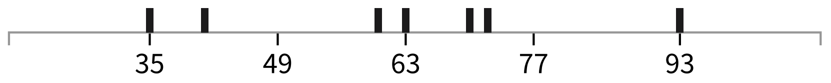

RT

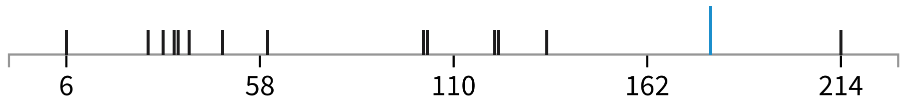

Drug Resistance Interpretation: PR

PI Major Resistance Mutations:	None
PI Accessory Resistance Mutations:	None
Other Mutations:	E35D, R41K, D60E, L63P, K70Q, I72V, I93L

Protease Inhibitors	
**atazanavir/r (ATV/r)**	Susceptible
**darunavir/r (DRV/r)**	Susceptible
**fosamprenavir/r (FPV/r)**	Susceptible
**indinavir/r (IDV/r)**	Susceptible
**lopinavir/r (LPV/r)**	Susceptible
**nelfinavir (NFV)**	Susceptible
**saquinavir/r (SQV/r)**	Susceptible
**tipranavir/r (TPV/r)**	Susceptible

Mutation Scoring: PR

PI	ATV/r	DRV/r	FPV/r	IDV/r	LPV/r	NFV	SQV/r	TPV/r
Total	0	0	0	0	0	0	0	0

Drug Resistance Interpretation: RT

NRTI Resistance Mutations:	None
NNRTI Resistance Mutations:	**V179D**
Other Mutations:	E6K, E28K, K32E, V35T, E36A, T39D, S48T, V60I, K102R, K103R, D121Y, K122E, I135K, F214L

Nucleoside Reverse Transcriptase Inhibitors

**abacavir (ABC)**	Susceptible
**zidovudine (AZT)**	Susceptible
**stavudine (D4T)**	Susceptible
**didanosine (DDI)**	Susceptible
**emtricitabine (FTC)**	Susceptible
**lamivudine (3TC)**	Susceptible
**tenofovir (TDF)**	Susceptible

Non-nucleoside Reverse Transcriptase Inhibitors

**efavirenz (EFV)**	Intermediate Resistance
**etravirine (ETR)**	Potential Low-Level Resistance
**nevirapine (NVP)**	Intermediate Resistance
**rilpivirine (RPV)**	Low-Level Resistance

RT Comments

NNRTI

- **V179D** is a polymorphic accessory NNRTI-selected mutation. It contributes low-levels reductions in susceptibility to each of the NNRTIs. The combination of **V179D** and K103R act synergistically to reduce NVP and EFV susceptibility. **V179D** has a low weight in the Tibotec ETR genotypic susceptibility score. V179E is a non-polymorphic mutation occasionally selected by NVP and EFV. V179E appears similar to **V179D** in its effects on NNRTIs. **V179D/E** do not appear to reduce the virological response to a first-line EFV-containing regimen.

Other

- **K103R** is a polymorphic mutation that alone has no effect on NNRTI susceptibility. However, in combination with V179D, it causes intermediate resistance to EFV and NVP.

Mutation Scoring: RT

NRTI	ABC	AZT	D4T	DDI	FTC	3TC	TDF
Total	0	0	0	0	0	0	0

NNRTI	EFV	ETR	NVP	RPV
V179D	10	10	10	10
K103R + V179D	20	0	20	15
Total	30	10	30	25

Sequence CQ13002447

Sequence Summary

Sequence includes PR:	codons 1 - 99
Sequence includes RT:	codons 1 - 241
Subtype:	CRF07_BC (2.84%)
PR SDRMs:	None
RT SDRMs:	None

Sequence Quality Assessment

PR

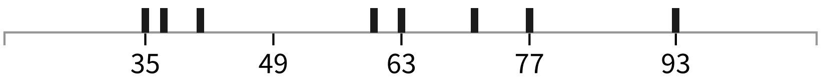

RT

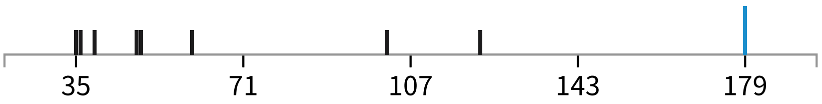

Drug Resistance Interpretation: PR

PI Major Resistance Mutations:	None
PI Accessory Resistance Mutations:	None
Other Mutations:	E35D, N37D, R41K, D60E, L63P, A71T, V77I, I93L

Protease Inhibitors	
**atazanavir/r (ATV/r)**	Susceptible
**darunavir/r (DRV/r)**	Susceptible
**fosamprenavir/r (FPV/r)**	Susceptible
**indinavir/r (IDV/r)**	Susceptible
**lopinavir/r (LPV/r)**	Susceptible
**nelfinavir (NFV)**	Susceptible
**saquinavir/r (SQV/r)**	Susceptible
**tipranavir/r (TPV/r)**	Susceptible

PR Comments

Other

- **A71V/T** are polymorphic, PI-selected accessory mutations that increase the replication of viruses with other PI-resistance mutations.

Mutation Scoring: PR

PI	ATV/r	DRV/r	FPV/r	IDV/r	LPV/r	NFV	SQV/r	TPV/r
Total	0	0	0	0	0	0	0	0

Drug Resistance Interpretation: RT

NRTI Resistance Mutations:	None
NNRTI Resistance Mutations:	**V179D**
Other Mutations:	V35T, E36A, T39D, S48T, K49R, V60I, K102R, K122E

Nucleoside Reverse Transcriptase Inhibitors

**abacavir (ABC)**	Susceptible
**zidovudine (AZT)**	Susceptible
**stavudine (D4T)**	Susceptible
**didanosine (DDI)**	Susceptible
**emtricitabine (FTC)**	Susceptible
**lamivudine (3TC)**	Susceptible
**tenofovir (TDF)**	Susceptible

Non-nucleoside Reverse Transcriptase Inhibitors

**efavirenz (EFV)**	Potential Low-Level Resistance
**etravirine (ETR)**	Potential Low-Level Resistance
**nevirapine (NVP)**	Potential Low-Level Resistance
**rilpivirine (RPV)**	Potential Low-Level Resistance

RT Comments

NNRTI

- **V179D** is a polymorphic accessory NNRTI-selected mutation. It contributes low-levels

reductions in susceptibility to each of the NNRTIs. The combination of **V179D** and K103R act synergistically to reduce NVP and EFV susceptibility. **V179D** has a low weight in the Tibotec ETR genotypic susceptibility score. V179E is a non-polymorphic mutation occasionally selected by NVP and EFV. V179E appears similar to **V179D** in its effects on NNRTIs. **V179D/E** do not appear to reduce the virological response to a first-line EFV-containing regimen.

Mutation Scoring: RT

NRTI	ABC	AZT	D4T	DDI	FTC	3TC	TDF
Total	0	0	0	0	0	0	0

NNRTI	EFV	ETR	NVP	RPV
V179D	10	10	10	10
Total	10	10	10	10

Sequence CQ13002852

Sequence Summary

Sequence includes PR:	codons 1 - 99
Sequence includes RT:	codons 1 - 241
Subtype:	B + C (3.04%)
PR SDRMs:	None
RT SDRMs:	None

Sequence Quality Assessment

PR

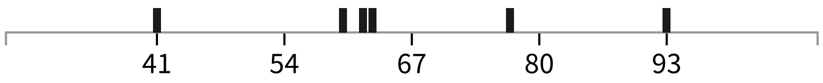

RT

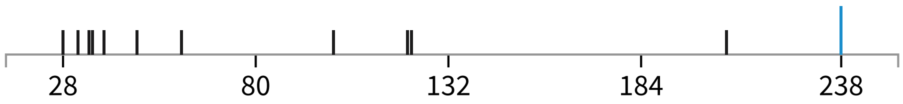

Drug Resistance Interpretation: PR

PI Major Resistance Mutations:	None
PI Accessory Resistance Mutations:	None
Other Mutations:	R41K, D60E, I62V, L63P, V77I, I93L

Protease Inhibitors	
**atazanavir/r (ATV/r)**	Susceptible
**darunavir/r (DRV/r)**	Susceptible
**fosamprenavir/r (FPV/r)**	Susceptible
**indinavir/r (IDV/r)**	Susceptible
**lopinavir/r (LPV/r)**	Susceptible
**nelfinavir (NFV)**	Susceptible
**saquinavir/r (SQV/r)**	Susceptible
**tipranavir/r (TPV/r)**	Susceptible

Mutation Scoring: PR

PI	ATV/r	DRV/r	FPV/r	IDV/r	LPV/r	NFV	SQV/r	TPV/r
Total	0	0	0	0	0	0	0	0

Drug Resistance Interpretation: RT

NRTI Resistance Mutations:	None
NNRTI Resistance Mutations:	**K238N**
Other Mutations:	E28K, K32E, V35T, E36A, T39D, S48T, V60I, K101R, D121Y, K122E, Q207H

Nucleoside Reverse Transcriptase Inhibitors

**abacavir (ABC)**	Susceptible
**zidovudine (AZT)**	Susceptible
**stavudine (D4T)**	Susceptible
**didanosine (DDI)**	Susceptible
**emtricitabine (FTC)**	Susceptible
**lamivudine (3TC)**	Susceptible
**tenofovir (TDF)**	Susceptible

Non-nucleoside Reverse Transcriptase Inhibitors

**efavirenz (EFV)**	Potential Low-Level Resistance
**etravirine (ETR)**	Susceptible
**nevirapine (NVP)**	Potential Low-Level Resistance
**rilpivirine (RPV)**	Susceptible

RT Comments

NNRTI

- K238T is a non-polymorphic mutation selected in patients receiving NVP and EFV. It usually occurs in combination with K103N. Alone it confers low/intermediate reductions in NVP and EFV susceptibility. **K238N** is a non-polymorphic accessory mutation that is also selected by NVP and EFV. It appears to have minimal, if any, effect on NNRTI susceptibility.

Mutation Scoring: RT

NRTI	ABC	AZT	D4T	DDI	FTC	3TC	TDF
Total	0	0	0	0	0	0	0

NNRTI	EFV	ETR	NVP	RPV
K238N	10	0	10	0
Total	10	0	10	0

Sequence CQ13003037

Sequence Summary

Sequence includes PR:	codons 1 - 99
Sequence includes RT:	codons 1 - 241
Subtype:	B + CRF01_AE (3.14%)
PR SDRMs:	None
RT SDRMs:	None

Sequence Quality Assessment

PR

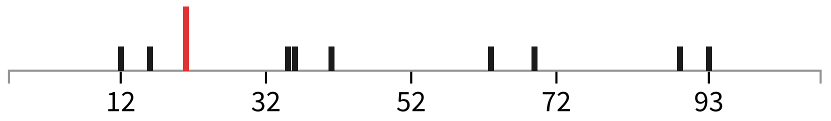

RT

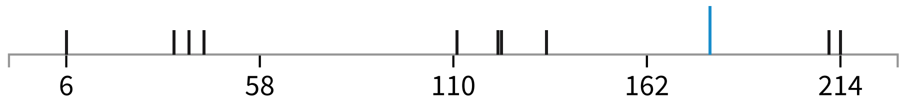

Drug Resistance Interpretation: PR

PI Major Resistance Mutations:	None
PI Accessory Resistance Mutations:	None
Other Mutations:	T12P, G16E, E21R, E35D, M36I, R41K, L63N, H69K, L89M, I93L

Protease Inhibitors	
**atazanavir/r (ATV/r)**	Susceptible
**darunavir/r (DRV/r)**	Susceptible
**fosamprenavir/r (FPV/r)**	Susceptible
**indinavir/r (IDV/r)**	Susceptible
**lopinavir/r (LPV/r)**	Susceptible
**nelfinavir (NFV)**	Susceptible
**saquinavir/r (SQV/r)**	Susceptible

**tipranavir/r (TPV/r)**	Susceptible

Mutation Scoring: PR

PI	ATV/r	DRV/r	FPV/r	IDV/r	LPV/r	NFV	SQV/r	TPV/r
Total	0	0	0	0	0	0	0	0

Drug Resistance Interpretation: RT

NRTI Resistance Mutations:	None
NNRTI Resistance Mutations:	**V179E**
Other Mutations:	E6D, V35T, T39K, K43E, V111I, K122E, D123S, I135R, R211K, F214L

Nucleoside Reverse Transcriptase Inhibitors

**abacavir (ABC)**	Susceptible
**zidovudine (AZT)**	Susceptible
**stavudine (D4T)**	Susceptible
**didanosine (DDI)**	Susceptible
**emtricitabine (FTC)**	Susceptible
**lamivudine (3TC)**	Susceptible
**tenofovir (TDF)**	Susceptible

Non-nucleoside Reverse Transcriptase Inhibitors

**efavirenz (EFV)**	Potential Low-Level Resistance
**etravirine (ETR)**	Potential Low-Level Resistance
**nevirapine (NVP)**	Potential Low-Level Resistance
**rilpivirine (RPV)**	Potential Low-Level Resistance

RT Comments

NNRTI

- V179D is a polymorphic accessory NNRTI-selected mutation. It contributes low-levels reductions in susceptibility to each of the NNRTIs. The combination of V179D and K103R act synergistically to reduce NVP and EFV susceptibility. V179D has a low weight in the Tibotec ETR genotypic susceptibility score. **V179E** is a non-polymorphic mutation occasionally selected by NVP and EFV. **V179E** appears similar to V179D in its

effects on NNRTIs. **V179D/E** do not appear to reduce the virological response to a first-line EFV-containing regimen.

Mutation Scoring: RT

NRTI	ABC	AZT	D4T	DDI	FTC	3TC	TDF
Total	0	0	0	0	0	0	0

NNRTI	EFV	ETR	NVP	RPV
V179E	10	10	10	10
Total	10	10	10	10

Sequence CQ13003228

Sequence Summary

Sequence includes PR:	codons 1 - 99
Sequence includes RT:	codons 1 - 241
Subtype:	B + C (6.08%)
PR SDRMs:	None
RT SDRMs:	None

Sequence Quality Assessment

PR

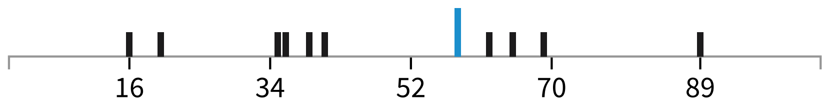

RT

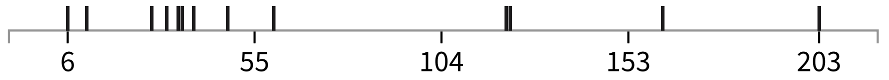

Drug Resistance Interpretation: PR

PI Major Resistance Mutations:	None
PI Accessory Resistance Mutations:	**Q58E**
Other Mutations:	G16E, K20R, E35D, M36I, P39S, R41K, I62V, E65D, H69K, L89M

Protease Inhibitors

**atazanavir/r (ATV/r)**	Susceptible
**darunavir/r (DRV/r)**	Susceptible
**fosamprenavir/r (FPV/r)**	Susceptible
**indinavir/r (IDV/r)**	Susceptible
**lopinavir/r (LPV/r)**	Susceptible
**nelfinavir (NFV)**	Potential Low-Level Resistance
**saquinavir/r (SQV/r)**	Susceptible

**tipranavir/r (TPV/r)**	Low-Level Resistance

PR Comments

PI Accessory

- **Q58E** is a non-polymorphic accessory PI-selected mutation associated with reduced susceptibility to TPV and possibly other PIs.

Other

- **K20R** is a highly polymorphic PI-selected accessory mutation.

Mutation Scoring: PR

PI	ATV/r	DRV/r	FPV/r	IDV/r	LPV/r	NFV	SQV/r	TPV/r
Q58E	0	0	0	0	0	10	0	15
Total	0	0	0	0	0	10	0	15

Drug Resistance Interpretation: RT

NRTI Resistance Mutations:	None
NNRTI Resistance Mutations:	None
Other Mutations:	E6D, K11T, E28K, K32E, V35T, E36A, T39D, S48T, V60I, D121Y, K122E, S162C, E203D

Nucleoside Reverse Transcriptase Inhibitors	
**abacavir (ABC)**	Susceptible
**zidovudine (AZT)**	Susceptible
**stavudine (D4T)**	Susceptible
**didanosine (DDI)**	Susceptible
**emtricitabine (FTC)**	Susceptible
**lamivudine (3TC)**	Susceptible
**tenofovir (TDF)**	Susceptible

Non-nucleoside Reverse Transcriptase Inhibitors	
**efavirenz (EFV)**	Susceptible
**etravirine (ETR)**	Susceptible

**nevirapine (NVP)**	Susceptible
**rilpivirine (RPV)**	Susceptible

Mutation Scoring: RT

NRTI	ABC	AZT	D4T	DDI	FTC	3TC	TDF
Total	0	0	0	0	0	0	0

NNRTI	EFV	ETR	NVP	RPV
Total	0	0	0	0

Sequence LY12003986

Sequence Summary

Sequence includes PR:	codons 1 - 99
Sequence includes RT:	codons 1 - 241
Subtype:	CRF55_01B (2.45%)
PR SDRMs:	None
RT SDRMs:	None

Sequence Quality Assessment

PR

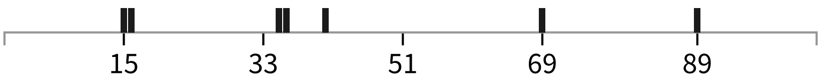

RT

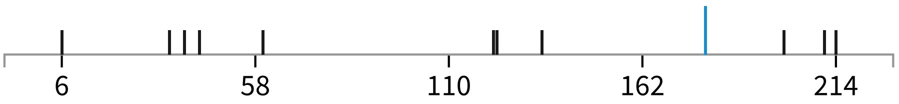

Drug Resistance Interpretation: PR

PI Major Resistance Mutations:	None
PI Accessory Resistance Mutations:	None
Other Mutations:	I15V, G16E, E35D, M36I, R41K, H69K, L89M

Protease Inhibitors	
**atazanavir/r (ATV/r)**	Susceptible
**darunavir/r (DRV/r)**	Susceptible
**fosamprenavir/r (FPV/r)**	Susceptible
**indinavir/r (IDV/r)**	Susceptible
**lopinavir/r (LPV/r)**	Susceptible
**nelfinavir (NFV)**	Susceptible
**saquinavir/r (SQV/r)**	Susceptible
**tipranavir/r (TPV/r)**	Susceptible

Mutation Scoring: PR

PI	ATV/r	DRV/r	FPV/r	IDV/r	LPV/r	NFV	SQV/r	TPV/r
Total	0	0	0	0	0	0	0	0

Drug Resistance Interpretation: RT

NRTI Resistance Mutations:	None
NNRTI Resistance Mutations:	**V179E**
Other Mutations:	E6D, V35T, T39K, K43E, V60I, K122E, D123S, I135R, T200I, R211K, F214L

Nucleoside Reverse Transcriptase Inhibitors

**abacavir (ABC)**	Susceptible
**zidovudine (AZT)**	Susceptible
**stavudine (D4T)**	Susceptible
**didanosine (DDI)**	Susceptible
**emtricitabine (FTC)**	Susceptible
**lamivudine (3TC)**	Susceptible
**tenofovir (TDF)**	Susceptible

Non-nucleoside Reverse Transcriptase Inhibitors

**efavirenz (EFV)**	Potential Low-Level Resistance
**etravirine (ETR)**	Potential Low-Level Resistance
**nevirapine (NVP)**	Potential Low-Level Resistance
**rilpivirine (RPV)**	Potential Low-Level Resistance

RT Comments

NNRTI

- V179D is a polymorphic accessory NNRTI-selected mutation. It contributes low-levels reductions in susceptibility to each of the NNRTIs. The combination of V179D and K103R act synergistically to reduce NVP and EFV susceptibility. V179D has a low weight in the Tibotec ETR genotypic susceptibility score. **V179E** is a non-polymorphic mutation occasionally selected by NVP and EFV. **V179E** appears similar to V179D in its effects on NNRTIs. **V179D/E** do not appear to reduce the virological response to a first-line EFV-containing regimen.

Mutation Scoring: RT

NRTI	ABC	AZT	D4T	DDI	FTC	3TC	TDF
Total	0	0	0	0	0	0	0

NNRTI	EFV	ETR	NVP	RPV
V179E	10	10	10	10
Total	10	10	10	10

Sequence LY12004233

Sequence Summary

Sequence includes PR:	codons 1 - 99
Sequence includes RT:	codons 1 - 241
Subtype:	B + C (3.24%)
PR SDRMs:	None
RT SDRMs:	G190E

Sequence Quality Assessment

PR

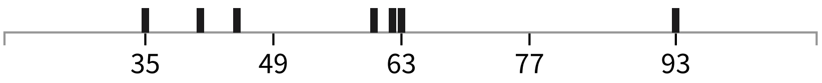

RT

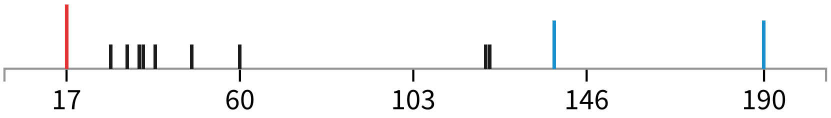

Drug Resistance Interpretation: PR

PI Major Resistance Mutations:	None
PI Accessory Resistance Mutations:	None
Other Mutations:	E35D, R41K, K45R, D60E, I62V, L63P, I93L

Protease Inhibitors	
**atazanavir/r (ATV/r)**	Susceptible
**darunavir/r (DRV/r)**	Susceptible
**fosamprenavir/r (FPV/r)**	Susceptible
**indinavir/r (IDV/r)**	Susceptible
**lopinavir/r (LPV/r)**	Susceptible
**nelfinavir (NFV)**	Susceptible
**saquinavir/r (SQV/r)**	Susceptible
**tipranavir/r (TPV/r)**	Susceptible

Mutation Scoring: PR

PI	ATV/r	DRV/r	FPV/r	IDV/r	LPV/r	NFV	SQV/r	TPV/r
Total	0	0	0	0	0	0	0	0

Drug Resistance Interpretation: RT

NRTI Resistance Mutations:	None
NNRTI Resistance Mutations:	**E138G**, **G190E**
Other Mutations:	D17N, E28K, K32E, V35T, E36A, T39N, S48T, V60I, D121Y, K122E

Nucleoside Reverse Transcriptase Inhibitors

**abacavir (ABC)**	Susceptible
**zidovudine (AZT)**	Susceptible
**stavudine (D4T)**	Susceptible
**didanosine (DDI)**	Susceptible
**emtricitabine (FTC)**	Susceptible
**lamivudine (3TC)**	Susceptible
**tenofovir (TDF)**	Susceptible

Non-nucleoside Reverse Transcriptase Inhibitors

**efavirenz (EFV)**	High-Level Resistance
**etravirine (ETR)**	Intermediate Resistance
**nevirapine (NVP)**	High-Level Resistance
**rilpivirine (RPV)**	High-Level Resistance

RT Comments

NNRTI

- **E138Q/G** are non-polymorphic accessory mutations frequently selected in patients receiving ETR and RPV and occasionally in patients receiving NVP and EFV. Preliminary data suggest that **E138Q/G** are associated with 2 to 3-fold reduced susceptibility to each of the NNRTIs.
- **G190E** is a non-polymorphic mutation that causes high-level resistance to each of the NNRTIs. G190Q is a less common non-polymorphic NNRTI-selected mutation that is associated with high-level NVP and EFV resistance and probable intermediate / high-level resistance to ETR and RPV.

Mutation Scoring: RT

NRTI	ABC	AZT	D4T	DDI	FTC	3TC	TDF
Total	0	0	0	0	0	0	0

NNRTI	EFV	ETR	NVP	RPV
E138G	10	10	10	15
G190E	60	45	60	60
Total	70	55	70	75

Sequence LY14003600

Sequence Summary

Sequence includes PR:	codons 1 - 99
Sequence includes RT:	codons 1 - 241
Subtype:	CRF55_01B (2.75%)
PR SDRMs:	None
RT SDRMs:	None

Sequence Quality Assessment

PR

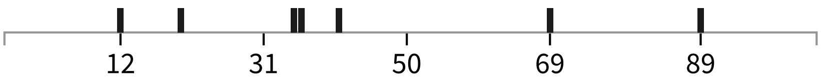

RT

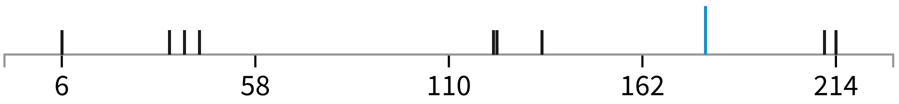

Drug Resistance Interpretation: PR

PI Major Resistance Mutations:	None
PI Accessory Resistance Mutations:	None
Other Mutations:	T12S, K20R, E35D, M36I, R41K, H69K, L89M

Protease Inhibitors	
**atazanavir/r (ATV/r)**	Susceptible
**darunavir/r (DRV/r)**	Susceptible
**fosamprenavir/r (FPV/r)**	Susceptible
**indinavir/r (IDV/r)**	Susceptible
**lopinavir/r (LPV/r)**	Susceptible
**nelfinavir (NFV)**	Susceptible
**saquinavir/r (SQV/r)**	Susceptible
**tipranavir/r (TPV/r)**	Susceptible

PR Comments

Other

- **K20R** is a highly polymorphic PI-selected accessory mutation.

Mutation Scoring: PR

PI	ATV/r	DRV/r	FPV/r	IDV/r	LPV/r	NFV	SQV/r	TPV/r
Total	0	0	0	0	0	0	0	0

Drug Resistance Interpretation: RT

NRTI Resistance Mutations:	None
NNRTI Resistance Mutations:	**V179E**
Other Mutations:	E6D, V35T, T39K, K43E, K122E, D123S, I135R, R211K, F214L

Nucleoside Reverse Transcriptase Inhibitors

**abacavir (ABC)**	Susceptible
**zidovudine (AZT)**	Susceptible
**stavudine (D4T)**	Susceptible
**didanosine (DDI)**	Susceptible
**emtricitabine (FTC)**	Susceptible
**lamivudine (3TC)**	Susceptible
**tenofovir (TDF)**	Susceptible

Non-nucleoside Reverse Transcriptase Inhibitors

**efavirenz (EFV)**	Potential Low-Level Resistance
**etravirine (ETR)**	Potential Low-Level Resistance
**nevirapine (NVP)**	Potential Low-Level Resistance
**rilpivirine (RPV)**	Potential Low-Level Resistance

RT Comments

NNRTI

- V179D is a polymorphic accessory NNRTI-selected mutation. It contributes low-levels

reductions in susceptibility to each of the NNRTIs. The combination of V179D and K103R act synergistically to reduce NVP and EFV susceptibility. V179D has a low weight in the Tibotec ETR genotypic susceptibility score. **V179E** is a non-polymorphic mutation occasionally selected by NVP and EFV. **V179E** appears similar to V179D in its effects on NNRTIs. **V179D/E** do not appear to reduce the virological response to a first-line EFV-containing regimen.

Mutation Scoring: RT

NRTI	ABC	AZT	D4T	DDI	FTC	3TC	TDF
Total	0	0	0	0	0	0	0

NNRTI	EFV	ETR	NVP	RPV
V179E	10	10	10	10
Total	10	10	10	10

Sequence MY14000058

Sequence Summary

Sequence includes PR:	codons 1 - 99
Sequence includes RT:	codons 1 - 241
Subtype:	CRF07_BC (2.84%)
PR SDRMs:	None
RT SDRMs:	None

Sequence Quality Assessment

PR

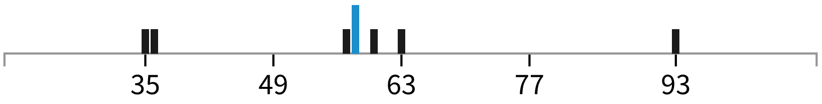

RT

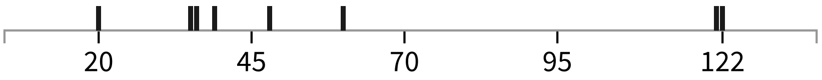

Drug Resistance Interpretation: PR

PI Major Resistance Mutations:	None
PI Accessory Resistance Mutations:	**Q58E**
Other Mutations:	E35D, M36I, R57K, D60E, L63P, I93L

Protease Inhibitors	
**atazanavir/r (ATV/r)**	Susceptible
**darunavir/r (DRV/r)**	Susceptible
**fosamprenavir/r (FPV/r)**	Susceptible
**indinavir/r (IDV/r)**	Susceptible
**lopinavir/r (LPV/r)**	Susceptible
**nelfinavir (NFV)**	Potential Low-Level Resistance
**saquinavir/r (SQV/r)**	Susceptible
**tipranavir/r (TPV/r)**	Low-Level Resistance

PR Comments

PI Accessory

- **Q58E** is a non-polymorphic accessory PI-selected mutation associated with reduced susceptibility to TPV and possibly other PIs.

Mutation Scoring: PR

PI	ATV/r	DRV/r	FPV/r	IDV/r	LPV/r	NFV	SQV/r	TPV/r
Q58E	0	0	0	0	0	10	0	15
Total	0	0	0	0	0	10	0	15

Drug Resistance Interpretation: RT

NRTI Resistance Mutations:	None
NNRTI Resistance Mutations:	None
Other Mutations:	K20R, V35M, E36A, T39D, S48T, V60I, D121Y, K122E

Nucleoside Reverse Transcriptase Inhibitors	
**abacavir (ABC)**	Susceptible
**zidovudine (AZT)**	Susceptible
**stavudine (D4T)**	Susceptible
**didanosine (DDI)**	Susceptible
**emtricitabine (FTC)**	Susceptible
**lamivudine (3TC)**	Susceptible
**tenofovir (TDF)**	Susceptible

Non-nucleoside Reverse Transcriptase Inhibitors	
**efavirenz (EFV)**	Susceptible
**etravirine (ETR)**	Susceptible
**nevirapine (NVP)**	Susceptible
**rilpivirine (RPV)**	Susceptible

Mutation Scoring: RT

NRTI	ABC	AZT	D4T	DDI	FTC	3TC	TDF
Total	0	0	0	0	0	0	0

NNRTI	EFV	ETR	NVP	RPV
Total	0	0	0	0

Sequence WS12000312

Sequence Summary

Sequence includes PR:	codons 1 - 99
Sequence includes RT:	codons 1 - 241
Subtype:	CRF55_01B (2.75%)
PR SDRMs:	None
RT SDRMs:	None

Sequence Quality Assessment

PR

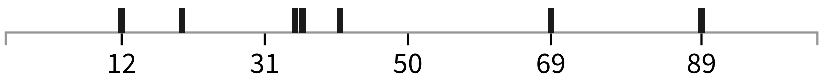

RT

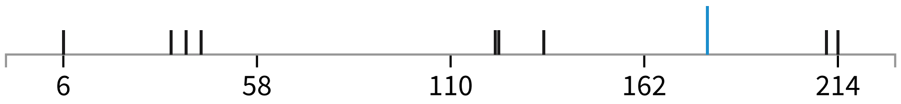

Drug Resistance Interpretation: PR

PI Major Resistance Mutations:	None
PI Accessory Resistance Mutations:	None
Other Mutations:	T12S, K20R, E35D, M36I, R41K, H69K, L89M

Protease Inhibitors	
**atazanavir/r (ATV/r)**	Susceptible
**darunavir/r (DRV/r)**	Susceptible
**fosamprenavir/r (FPV/r)**	Susceptible
**indinavir/r (IDV/r)**	Susceptible
**lopinavir/r (LPV/r)**	Susceptible
**nelfinavir (NFV)**	Susceptible
**saquinavir/r (SQV/r)**	Susceptible
**tipranavir/r (TPV/r)**	Susceptible

PR Comments

Other

- **K20R** is a highly polymorphic PI-selected accessory mutation.

Mutation Scoring: PR

PI	ATV/r	DRV/r	FPV/r	IDV/r	LPV/r	NFV	SQV/r	TPV/r
Total	0	0	0	0	0	0	0	0

Drug Resistance Interpretation: RT

NRTI Resistance Mutations:	None
NNRTI Resistance Mutations:	**V179E**
Other Mutations:	E6D, V35T, T39K, K43E, K122E, D123S, I135R, R211K, F214L

Nucleoside Reverse Transcriptase Inhibitors

**abacavir (ABC)**	Susceptible
**zidovudine (AZT)**	Susceptible
**stavudine (D4T)**	Susceptible
**didanosine (DDI)**	Susceptible
**emtricitabine (FTC)**	Susceptible
**lamivudine (3TC)**	Susceptible
**tenofovir (TDF)**	Susceptible

Non-nucleoside Reverse Transcriptase Inhibitors

**efavirenz (EFV)**	Potential Low-Level Resistance
**etravirine (ETR)**	Potential Low-Level Resistance
**nevirapine (NVP)**	Potential Low-Level Resistance
**rilpivirine (RPV)**	Potential Low-Level Resistance

RT Comments

NNRTI

- V179D is a polymorphic accessory NNRTI-selected mutation. It contributes low-levels

reductions in susceptibility to each of the NNRTIs. The combination of V179D and K103R act synergistically to reduce NVP and EFV susceptibility. V179D has a low weight in the Tibotec ETR genotypic susceptibility score. **V179E** is a non-polymorphic mutation occasionally selected by NVP and EFV. **V179E** appears similar to V179D in its effects on NNRTIs. **V179D/E** do not appear to reduce the virological response to a first-line EFV-containing regimen.

Mutation Scoring: RT

NRTI	ABC	AZT	D4T	DDI	FTC	3TC	TDF
Total	0	0	0	0	0	0	0

NNRTI	EFV	ETR	NVP	RPV
V179E	10	10	10	10
Total	10	10	10	10

Sequence WS12000334

Sequence Summary

Sequence includes PR:	codons 1 - 99
Sequence includes RT:	codons 1 - 241
Subtype:	B (7.16%)
PR SDRMs:	M46I
RT SDRMs:	None

Sequence Quality Assessment

PR

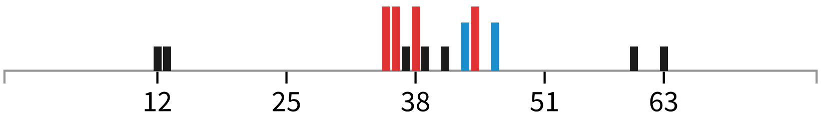

RT

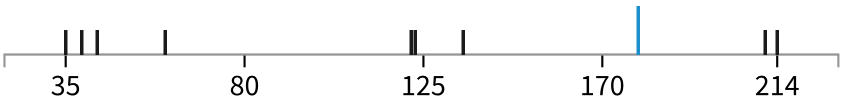

- **Note**: There are 4 unusual mutations in PR: E35V,M36Q,L38Q,P44Y.

Drug Resistance Interpretation: PR

PI Major Resistance Mutations:	**M46I**
PI Accessory Resistance Mutations:	**K43T**
Other Mutations:	T12P, I13V, E35V, M36Q, N37K, L38Q, P39A, R41P, P44Y, D60E, L63P

Protease Inhibitors

**atazanavir/r (ATV/r)**	Potential Low-Level Resistance
**darunavir/r (DRV/r)**	Susceptible
**fosamprenavir/r (FPV/r)**	Potential Low-Level Resistance
**indinavir/r (IDV/r)**	Potential Low-Level Resistance

**lopinavir/r (LPV/r)**	Potential Low-Level Resistance
**nelfinavir (NFV)**	Intermediate Resistance
**saquinavir/r (SQV/r)**	Potential Low-Level Resistance
**tipranavir/r (TPV/r)**	Low-Level Resistance

PR Comments

PI Major

- **M46I/L** are relatively non-polymorphic PI-selected mutations. In combination with other PI-resistance mutations, they are associated with reduced susceptibility to each of the PIs except DRV.

PI Accessory

- **K43T** is a non-polymorphic PI-selected accessory mutation. **K43T** is included in the Boehringer-Ingelheim TPV genotypic susceptibility score.

Mutation Scoring: PR

PI	ATV/r	DRV/r	FPV/r	IDV/r	LPV/r	NFV	SQV/r	TPV/r
M46I	10	0	10	10	10	30	10	5
K43T	0	0	0	0	0	10	0	10
Total	10	0	10	10	10	40	10	15

Drug Resistance Interpretation: RT

NRTI Resistance Mutations:	None
NNRTI Resistance Mutations:	**V179E**
Other Mutations:	V35T, T39K, K43E, V60I, K122E, D123S, I135R, R211K, F214L

Nucleoside Reverse Transcriptase Inhibitors

**abacavir (ABC)**	Susceptible
**zidovudine (AZT)**	Susceptible
**stavudine (D4T)**	Susceptible
**didanosine (DDI)**	Susceptible
**emtricitabine (FTC)**	Susceptible

**lamivudine (3TC)**	Susceptible
**tenofovir (TDF)**	Susceptible

Non-nucleoside Reverse Transcriptase Inhibitors

**efavirenz (EFV)**	Potential Low-Level Resistance
**etravirine (ETR)**	Potential Low-Level Resistance
**nevirapine (NVP)**	Potential Low-Level Resistance
**rilpivirine (RPV)**	Potential Low-Level Resistance

RT Comments

NNRTI

- V179D is a polymorphic accessory NNRTI-selected mutation. It contributes low-levels reductions in susceptibility to each of the NNRTIs. The combination of V179D and K103R act synergistically to reduce NVP and EFV susceptibility. V179D has a low weight in the Tibotec ETR genotypic susceptibility score. **V179E** is a non-polymorphic mutation occasionally selected by NVP and EFV. **V179E** appears similar to V179D in its effects on NNRTIs. **V179D/E** do not appear to reduce the virological response to a first-line EFV-containing regimen.

Mutation Scoring: RT

NRTI	ABC	AZT	D4T	DDI	FTC	3TC	TDF
Total	0	0	0	0	0	0	0

NNRTI	EFV	ETR	NVP	RPV
V179E	10	10	10	10
Total	10	10	10	10

Sequence WS12000352

Sequence Summary

Sequence includes PR:	codons 1 - 99
Sequence includes RT:	codons 1 - 241
Subtype:	CRF07_BC (2.75%)
PR SDRMs:	None
RT SDRMs:	None

Sequence Quality Assessment

PR

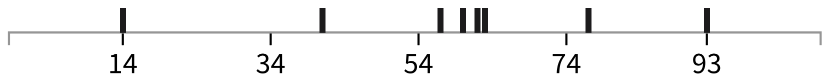

RT

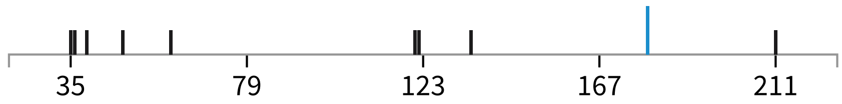

Drug Resistance Interpretation: PR

PI Major Resistance Mutations:	None
PI Accessory Resistance Mutations:	None
Other Mutations:	K14R, R41K, R57K, D60E, I62V, L63P, V77I, I93L

Protease Inhibitors	
**atazanavir/r (ATV/r)**	Susceptible
**darunavir/r (DRV/r)**	Susceptible
**fosamprenavir/r (FPV/r)**	Susceptible
**indinavir/r (IDV/r)**	Susceptible
**lopinavir/r (LPV/r)**	Susceptible
**nelfinavir (NFV)**	Susceptible
**saquinavir/r (SQV/r)**	Susceptible
**tipranavir/r (TPV/r)**	Susceptible

Mutation Scoring: PR

PI	ATV/r	DRV/r	FPV/r	IDV/r	LPV/r	NFV	SQV/r	TPV/r
Total	0	0	0	0	0	0	0	0

Drug Resistance Interpretation: RT

NRTI Resistance Mutations: None
NNRTI Resistance Mutations: **V179D**
Other Mutations: V35T, E36A, T39D, S48T, V60I, D121H, K122E, I135T, R211K

Nucleoside Reverse Transcriptase Inhibitors

**abacavir (ABC)**	Susceptible
**zidovudine (AZT)**	Susceptible
**stavudine (D4T)**	Susceptible
**didanosine (DDI)**	Susceptible
**emtricitabine (FTC)**	Susceptible
**lamivudine (3TC)**	Susceptible
**tenofovir (TDF)**	Susceptible

Non-nucleoside Reverse Transcriptase Inhibitors

**efavirenz (EFV)**	Potential Low-Level Resistance
**etravirine (ETR)**	Potential Low-Level Resistance
**nevirapine (NVP)**	Potential Low-Level Resistance
**rilpivirine (RPV)**	Potential Low-Level Resistance

RT Comments

NNRTI

- **V179D** is a polymorphic accessory NNRTI-selected mutation. It contributes low-levels reductions in susceptibility to each of the NNRTIs. The combination of **V179D** and K103R act synergistically to reduce NVP and EFV susceptibility. **V179D** has a low weight in the Tibotec ETR genotypic susceptibility score. V179E is a non-polymorphic mutation occasionally selected by NVP and EFV. V179E appears similar to **V179D** in its effects on NNRTIs. **V179D/E** do not appear to reduce the virological response to a first-line EFV-containing regimen.

Mutation Scoring: RT

NRTI	ABC	AZT	D4T	DDI	FTC	3TC	TDF
Total	0	0	0	0	0	0	0

NNRTI	EFV	ETR	NVP	RPV
V179D	10	10	10	10
Total	10	10	10	10

Sequence ws12001012

Sequence Summary

Sequence includes PR:	codons 1 - 99
Sequence includes RT:	codons 1 - 241
Subtype:	CRF01_AE (4.12%)
PR SDRMs:	None
RT SDRMs:	None

Sequence Quality Assessment

PR

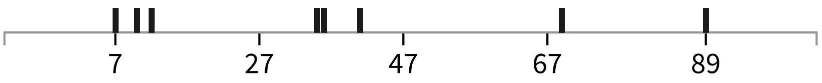

RT

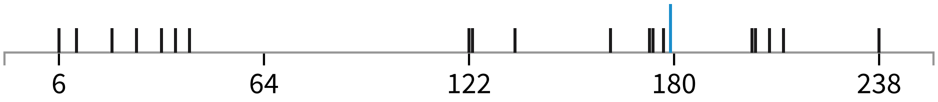

Drug Resistance Interpretation: PR

PI Major Resistance Mutations:	None
PI Accessory Resistance Mutations:	None
Other Mutations:	Q7R, L10I, T12A, E35D, M36I, R41K, H69K, L89M

Protease Inhibitors	
**atazanavir/r (ATV/r)**	Susceptible
**darunavir/r (DRV/r)**	Susceptible
**fosamprenavir/r (FPV/r)**	Susceptible
**indinavir/r (IDV/r)**	Susceptible
**lopinavir/r (LPV/r)**	Susceptible
**nelfinavir (NFV)**	Susceptible
**saquinavir/r (SQV/r)**	Susceptible
**tipranavir/r (TPV/r)**	Susceptible

PR Comments

Other

- **L10I/V** are polymorphic, PI-selected accessory mutations that increase the replication of viruses with other PI-resistance mutations.

Mutation Scoring: PR

PI	ATV/r	DRV/r	FPV/r	IDV/r	LPV/r	NFV	SQV/r	TPV/r
Total	0	0	0	0	0	0	0	0

Drug Resistance Interpretation: RT

NRTI Resistance Mutations:	None
NNRTI Resistance Mutations:	**V179D**
Other Mutations:	E6D, K11T, V21I, E28A, V35T, T39R, K43E, K122E, D123S, I135V, S162C, K173S, Q174K, D177G, I202V, E203D, Q207A, R211S, K238R

Nucleoside Reverse Transcriptase Inhibitors

**abacavir (ABC)**	Susceptible
**zidovudine (AZT)**	Susceptible
**stavudine (D4T)**	Susceptible
**didanosine (DDI)**	Susceptible
**emtricitabine (FTC)**	Susceptible
**lamivudine (3TC)**	Susceptible
**tenofovir (TDF)**	Susceptible

Non-nucleoside Reverse Transcriptase Inhibitors

**efavirenz (EFV)**	Potential Low-Level Resistance
**etravirine (ETR)**	Potential Low-Level Resistance
**nevirapine (NVP)**	Potential Low-Level Resistance
**rilpivirine (RPV)**	Potential Low-Level Resistance

RT Comments

NNRTI

- **V179D** is a polymorphic accessory NNRTI-selected mutation. It contributes low-levels reductions in susceptibility to each of the NNRTIs. The combination of **V179D** and K103R act synergistically to reduce NVP and EFV susceptibility. **V179D** has a low weight in the Tibotec ETR genotypic susceptibility score. V179E is a non-polymorphic mutation occasionally selected by NVP and EFV. V179E appears similar to **V179D** in its effects on NNRTIs. **V179D/E** do not appear to reduce the virological response to a first-line EFV-containing regimen.

Other

- **K238R** is a common polymorphism that does not reduce NNRTI susceptibility.

Mutation Scoring: RT

NRTI	ABC	AZT	D4T	DDI	FTC	3TC	TDF
Total	0	0	0	0	0	0	0

NNRTI	EFV	ETR	NVP	RPV
V179D	10	10	10	10
Total	10	10	10	10

Sequence WS12001503

Sequence Summary

Sequence includes PR:	codons 1 - 99
Sequence includes RT:	codons 1 - 241
Subtype:	CRF55_01B (2.25%)
PR SDRMs:	None
RT SDRMs:	None

Sequence Quality Assessment

PR

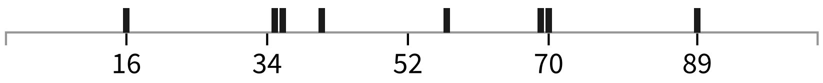

RT

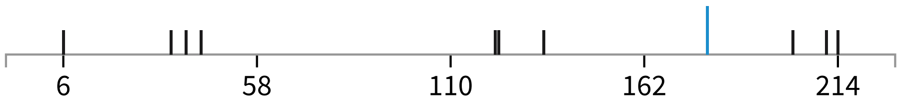

Drug Resistance Interpretation: PR

PI Major Resistance Mutations:	None
PI Accessory Resistance Mutations:	None
Other Mutations:	G16E, E35D, M36I, R41K, R57K, H69K, K70R, L89M

Protease Inhibitors	
**atazanavir/r (ATV/r)**	Susceptible
**darunavir/r (DRV/r)**	Susceptible
**fosamprenavir/r (FPV/r)**	Susceptible
**indinavir/r (IDV/r)**	Susceptible
**lopinavir/r (LPV/r)**	Susceptible
**nelfinavir (NFV)**	Susceptible
**saquinavir/r (SQV/r)**	Susceptible
**tipranavir/r (TPV/r)**	Susceptible

Mutation Scoring: PR

PI	ATV/r	DRV/r	FPV/r	IDV/r	LPV/r	NFV	SQV/r	TPV/r
Total	0	0	0	0	0	0	0	0

Drug Resistance Interpretation: RT

NRTI Resistance Mutations:	None
NNRTI Resistance Mutations:	**V179E**
Other Mutations:	E6D, V35T, T39K, K43E, K122E, D123S, I135R, I202V, R211K, F214L

Nucleoside Reverse Transcriptase Inhibitors

**abacavir (ABC)**	Susceptible
**zidovudine (AZT)**	Susceptible
**stavudine (D4T)**	Susceptible
**didanosine (DDI)**	Susceptible
**emtricitabine (FTC)**	Susceptible
**lamivudine (3TC)**	Susceptible
**tenofovir (TDF)**	Susceptible

Non-nucleoside Reverse Transcriptase Inhibitors

**efavirenz (EFV)**	Potential Low-Level Resistance
**etravirine (ETR)**	Potential Low-Level Resistance
**nevirapine (NVP)**	Potential Low-Level Resistance
**rilpivirine (RPV)**	Potential Low-Level Resistance

RT Comments

NNRTI

- V179D is a polymorphic accessory NNRTI-selected mutation. It contributes low-levels reductions in susceptibility to each of the NNRTIs. The combination of V179D and K103R act synergistically to reduce NVP and EFV susceptibility. V179D has a low weight in the Tibotec ETR genotypic susceptibility score. **V179E** is a non-polymorphic mutation occasionally selected by NVP and EFV. **V179E** appears similar to V179D in its effects on NNRTIs. **V179D/E** do not appear to reduce the virological response to a first-line EFV-containing regimen.

Mutation Scoring: RT

NRTI	ABC	AZT	D4T	DDI	FTC	3TC	TDF
Total	0	0	0	0	0	0	0

NNRTI	EFV	ETR	NVP	RPV
V179E	10	10	10	10
Total	10	10	10	10

Sequence WS13003163

Sequence Summary

Sequence includes PR:	codons 1 - 99
Sequence includes RT:	codons 1 - 241
Subtype:	B + C (3.53%)
PR SDRMs:	None
RT SDRMs:	None

Sequence Quality Assessment

PR

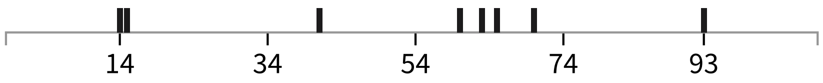

RT

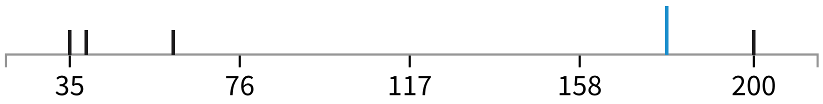

Drug Resistance Interpretation: PR

PI Major Resistance Mutations:	None
PI Accessory Resistance Mutations:	None
Other Mutations:	K14R, I15V, R41K, D60E, L63P, E65D, K70T, I93L

Protease Inhibitors	
**atazanavir/r (ATV/r)**	Susceptible
**darunavir/r (DRV/r)**	Susceptible
**fosamprenavir/r (FPV/r)**	Susceptible
**indinavir/r (IDV/r)**	Susceptible
**lopinavir/r (LPV/r)**	Susceptible
**nelfinavir (NFV)**	Susceptible
**saquinavir/r (SQV/r)**	Susceptible
**tipranavir/r (TPV/r)**	Susceptible

Mutation Scoring: PR

PI	ATV/r	DRV/r	FPV/r	IDV/r	LPV/r	NFV	SQV/r	TPV/r
Total	0	0	0	0	0	0	0	0

Drug Resistance Interpretation: RT

NRTI Resistance Mutations:	None
NNRTI Resistance Mutations:	**V179D**
Other Mutations:	V35K, T39D, V60I, T200A

Nucleoside Reverse Transcriptase Inhibitors

**abacavir (ABC)**	Susceptible
**zidovudine (AZT)**	Susceptible
**stavudine (D4T)**	Susceptible
**didanosine (DDI)**	Susceptible
**emtricitabine (FTC)**	Susceptible
**lamivudine (3TC)**	Susceptible
**tenofovir (TDF)**	Susceptible

Non-nucleoside Reverse Transcriptase Inhibitors

**efavirenz (EFV)**	Potential Low-Level Resistance
**etravirine (ETR)**	Potential Low-Level Resistance
**nevirapine (NVP)**	Potential Low-Level Resistance
**rilpivirine (RPV)**	Potential Low-Level Resistance

RT Comments

NNRTI

- **V179D** is a polymorphic accessory NNRTI-selected mutation. It contributes low-levels reductions in susceptibility to each of the NNRTIs. The combination of **V179D** and K103R act synergistically to reduce NVP and EFV susceptibility. **V179D** has a low weight in the Tibotec ETR genotypic susceptibility score. V179E is a non-polymorphic mutation occasionally selected by NVP and EFV. V179E appears similar to **V179D** in its effects on NNRTIs. **V179D/E** do not appear to reduce the virological response to a first-line EFV-containing regimen.

Mutation Scoring: RT

NRTI	ABC	AZT	D4T	DDI	FTC	3TC	TDF
Total	0	0	0	0	0	0	0

NNRTI	EFV	ETR	NVP	RPV
V179D	10	10	10	10
Total	10	10	10	10

© 1998 - 2017. All Rights Reserved. Questions? Contact HIVdb <rshafer@stanford.edu, syrhee@stanford.edu, philiptz@stanford.edu>.
